# Mitochondrial *O*-GlcNAc Transferase Interacts with and Modifies Many Proteins and Its Up-Regulation Affects Mitochondrial Function and Cellular Energy Homeostasis

**DOI:** 10.3390/cancers13122956

**Published:** 2021-06-12

**Authors:** Paweł Jóźwiak, Piotr Ciesielski, Piotr K. Zakrzewski, Karolina Kozal, Joanna Oracz, Grażyna Budryn, Dorota Żyżelewicz, Stéphanie Flament, Anne-Sophie Vercoutter-Edouart, Fabrice Bray, Tony Lefebvre, Anna Krześlak

**Affiliations:** 1 Department of Cytobiochemistry, Faculty of Biology and Environmental Protection, University of Lodz, 90-236 Lodz, Poland; piotr.ciesielski@biol.uni.lodz.pl (P.C.); piotr.zakrzewski@biol.uni.lodz.pl (P.K.Z.); karolina.kozal@edu.uni.lodz.pl (K.K.); anna.krzeslak@biol.uni.lodz.pl (A.K.); 2 Institute of Food Technology and Analysis, Faculty of Biotechnology and Food Sciences, Lodz University of Technology, 90-924 Lodz, Poland; joanna.oracz@p.lodz.pl (J.O.); grazyna.budryn@p.lodz.pl (G.B.); dorota.zyzelewicz@p.lodz.pl (D.Ż.); 3 Institut Eugène-Michel Chevreul, CNRS, MSAP USR 3290, FR 3688 FRABIO, FR 2638, Université de Lille, 59000 Lille, France; stephanie.flament@univ-lille.fr (S.F.); fabrice.bray@univ-lille.fr (F.B.); 4 Unité de Glycobiologie Structurale et Fonctionnelle, CNRS, UMR 8576, UGSF, Université de Lille, 59000 Lille, France; anne-sophie.vercoutter@univ-lille.fr (A.-S.V.-E.); tony.lefebvre@univ-lille.fr (T.L.)

**Keywords:** mOGT, *O*-GlcNAc, mitochondria, glucose, energy metabolism, breast cancer

## Abstract

**Simple Summary:**

*O*-GlcNAcylation is a dynamic post-translational modification of proteins involved in the control of intracellular signaling pathways in response to changes in nutrient availability, especially glucose concentration. To date, most research has focused on *O*-GlcNAcylation of proteins by the nuclear-cytoplasmic isoform of *O*-GlcNAc transferase (ncOGT), while the role of mitochondrial OGT (mOGT) and its effect on *O*-GlcNAcylation of mitochondrial proteins are poorly understood. The aim of our study was to investigate the effect of mOGT on *O*-GlcNAcylation of mitochondrial proteins, mitochondrial function, and energy metabolism of breast cancer cells. Herein, we used two independent proteomics-based approaches to identify mOGT-interacting partners and proteins modified by mOGT. Based on our findings, we propose that *O*-GlcNAcylation of proteins by mOGT is a part of the mechanism by which glucose affects mitochondrial function and cellular bioenergetics.

**Abstract:**

*O*-GlcNAcylation is a cell glucose sensor. The addition of *O*-GlcNAc moieties to target protein is catalyzed by the *O*-Linked *N*-acetylglucosamine transferase (OGT). OGT is encoded by a single gene that yields differentially spliced OGT isoforms. One of them is targeted to mitochondria (mOGT). Although the impact of *O*-GlcNAcylation on cancer cells biology is well documented, mOGT’s role remains poorly investigated. We performed studies using breast cancer cells with up-regulated mOGT or its catalytic inactive mutant to identify proteins specifically modified by mOGT. Proteomic approaches included isolation of mOGT protein partners and *O*-GlcNAcylated proteins from mitochondria-enriched fraction followed by their analysis by mass spectrometry. Moreover, we analyzed the impact of mOGT dysregulation on mitochondrial activity and cellular metabolism using a variety of biochemical assays. We found that mitochondrial OGT expression is glucose-dependent. Elevated mOGT expression affected the mitochondrial transmembrane potential and increased intramitochondrial ROS generation. Moreover, mOGT up-regulation caused a decrease in cellular ATP level. We identified many mitochondrial proteins as mOGT substrates. Most of these proteins are localized in the mitochondrial matrix and the inner mitochondrial membrane and participate in mitochondrial respiration, fatty acid metabolism, transport, translation, apoptosis, and mtDNA processes. Our findings suggest that mOGT interacts with and modifies many mitochondrial proteins, and its dysregulation affects cellular bioenergetics and mitochondria function.

## 1. Introduction

Cancer cells undergo significant metabolic changes that allow rapid growth and proliferation. The classic bioenergetic phenotype of cancer cells usually displays an enhanced rate of glycolysis along with inhibition of oxidative phosphorylation even in the presence of oxygen, a phenomenon known as “Warburg effect”. It is well-known that these metabolic alterations are strongly dependent on glucose consumption and utilization [1,2].

**Figure 1:**
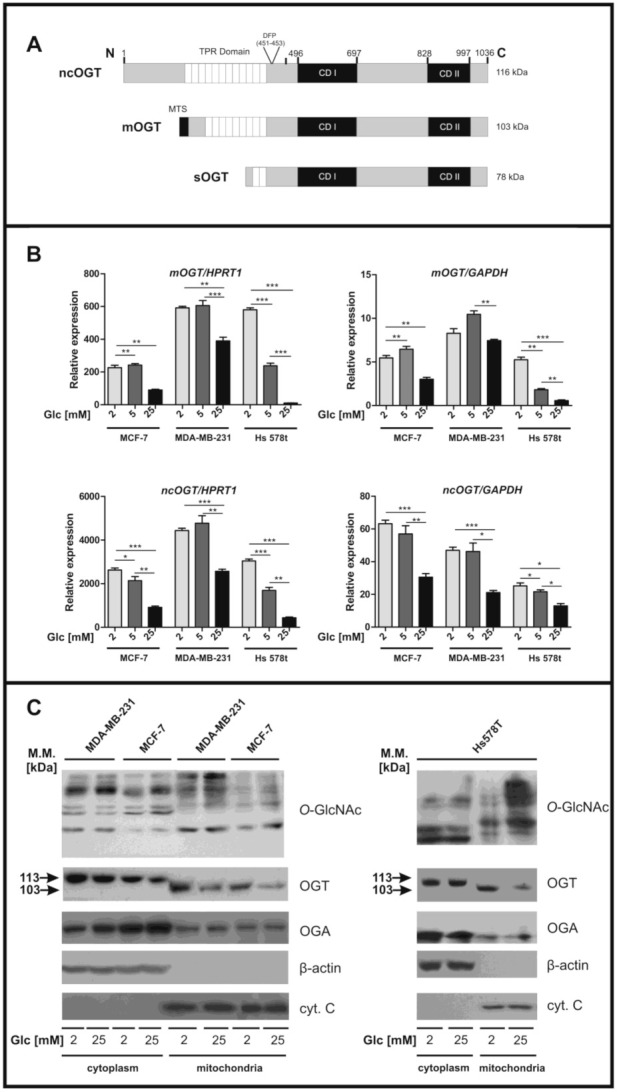
Glucose concentration alters the expression of OGT isoforms and *O*-GlcNAc levels in mitochondria. (**A**) Schematic structure of OGT isoforms. (**B**) *mOGT* and *ncOGT* transcript levels were determined using RT-qPCR, and the results were referenced to *HPRT1* and *GAPDH* housekeeping genes mRNA. The bars represent mean +/− SD from three independent experiments in duplicate. (**C**) *O*-GlcNAc, OGT, and OGA levels in cytoplasmic (30 μg of proteins per line) and mitochondrial (60 μg of proteins per line) fractions of breast cancer cells. For cytoplasmic extract, β-actin was used as a loading control, whereas cytochrome C was used as a marker of mitochondria-enriched fraction. * indicates significance; *p* < 0.05; ** *p* < 0.01; *** *p* < 0.001. Glc, glucose. (The original western blot images are shown in the File S1).

Within the cell, glucose is mainly metabolized into pyruvate through glycolysis, but a fraction of approximately 2–4% enters the hexosamine biosynthetic pathway (HBP). The end product of the HBP is the nucleotide sugar uridine diphosphate N-acetylglucosamine (UDP-GlcNAc), used for many reactions as a sugar donor, including β-*O*-linked N-acetylglucosaminylation (*O*-GlcNAcylation) [3]. All *O*-GlcNAc-modified proteins are phosphoproteins and sometimes *O*-GlcNAc and *O*-phosphate moieties compete for the same or neighboring site [4]. The interplay between the two post-translational modifications can determine target protein function such as subcellular transport and localization, complex formation, or enzyme activity; therefore, the reciprocal relationship between these modifications plays a significant role in the regulation of multiple biological processes [5]. Dynamic *O*-GlcNAc cycling is regulated by two key enzymes: the *O*-linked β-N-acetyl-D-glucosaminyltransferase (OGT) is responsible for the addition of a single GlcNAc residue to proteins, whereas *O*-linked β-N-acetyl-D-glucosaminidase (OGA) catalyzes the removal of the GlcNAc moiety [6,7]. In humans, there is only a single *OGT* gene located on chromosome Xq13.1, a region associated with Parkinson’s disease [8]. To date, three functional isoforms of the OGT enzyme, arising possibly by alternative splicing or alternative promoters, have been identified. The longest (~116 kDa, ncOGT) and the shortest (~78 kDa, sOGT) isoforms are located in both the nucleus and the cytoplasm (Figure 1A). The third isoform (~103 kDa) contains a mitochondrial targeting sequence (MTS) at its N-terminus and has been referred to as mitochondrial OGT (mOGT) [9]. 

Over the last two decades, there has been solid evidence supporting a pivotal role of *O*-GlcNAcylation in cancer cell biology. The results of many studies suggest that increased expression of OGT and hyper-*O*-GlcNAcylation are the universal features of cancers, including breast cancer [10,11]. Accumulating evidence suggests that OGT may act as a nutrient sensor that links HBP to oncogenic signaling and regulation of factors involved in glucose metabolism and cancer progression. *O*-GlcNAc signaling has been proven essential for the onset, progression, and metastasis of breast cancer [12,13]. It has been shown that decreased *O*-GlcNAcylation as a result of reduced expression of OGT by siRNA causes profound changes in the proteome of MCF-7 breast cancer cells. The gel-free quantitative proteomics coupled with LC–MS/MS analysis allows identifying proteins affected by *O*-GlcNAc inhibition, which may have important roles in cancer metastasis. Among proteins whose expression was affected by *O*-GlcNAc changes, proteins involved in cellular metabolism, cellular localization, stress responses, and gene expression were identified [14]. In spite of *O*-GlcNAc significance in breast cancer cells, the impact of *O*-GlcNAc cycling enzymes on mitochondria function was not yet investigated. However, there are several examples of roles of *O*-GlcNAc in other cancerous and non-cancerous cell types. Elevated *O*-GlcNAcylation of respiratory chain complex I and III proteins is associated with impaired mitochondrial function in cardiomyocytes under hyperglycemic conditions [15]. Furthermore, *O*-GlcNAc cycling modulates activity and translocation of the dynamin-related protein 1 (DRP1), whose activation is associated with the loss of membrane potential and mitochondria fragmentation [16]. A recent study performed on rat purified cardiac mitochondria revealed that among the 88 identified *O*-GlcNAcylated mitochondrial candidate proteins, nearly half of them are components of the oxidative phosphorylation system [17]. In addition, the identification of the pyrimidine nucleotide carrier 1 (pnc1) as a UDP-GlcNAc transporter reinforces the view that mitochondria possess the machinery for switching *O*-GlcNAc cycling on and off [18]. Until now, the studies concerning the links between *O*-GlcNAcylation and cancer biology have been concentrated on the ncOGT activity, whereas the reports related to the role of mOGT are very limited. Trapannone et al. [19] suggested that ncOGT was sufficient for *O*-GlcNAc moieties attachment to mitochondrial proteins. However, the identification of some *O*-GlcNAcylated proteins encoded by mitochondrial DNA (cytochrome oxidase 1; MTCO1; cytochrome oxidase 2; COX2 and NADH:ubiquinone oxidoreductase core subunit 4; MT-ND4) raises the possibility that the mOGT isoform is required for their modification. Depletion of mOGT by siRNA allowed the identification of four putative mOGT’s protein substrates [15,16,17]. The vast majority of mitochondrial proteins are encoded by nuclear genes and transported into mitochondria after synthesis in the cytosol. Therefore, part of proteins residing in mitochondria could be *O*-GlcNAcylated by the ncOGT prior to translocation to mitochondria. However, early reports clearly showed that some proteins, such as Nup62 and casein kinase II, were glycosylated by both ncOGT and mOGT, while other ones, such as yes kinase, were specifically modified by mOGT [20]. The substrate selectivity most likely occurs due to the different number of tetratricopeptide repeats (TPR) in the N-terminal region of OGT isoforms [21]. Therefore, mOGT may have different effects on mitochondria function than ncOGT; as an example, mOGT, overexpressed in cancer cell lines, could induce apoptosis probably as a pro-apoptotic partner. However, the impact of mOGT on mitochondrial homeostasis and cellular physiology still remains substantially unknown. 

Thus, in this study, in order to bring closer the significance of mOGT in cancer cells, we identified mOGT protein substrates and investigated the effect of mOGT dysregulation on mitochondrial activity of breast cancer cells.

## 2. Materials and Methods

### 2.1. Reagents and Antibodies

Chemicals were obtained from Sigma-Aldrich (St. Louis, MO, USA) except when specified. Cell culture reagents and materials were purchased from Invitrogen (Carlsbad, CA, USA), Cytogen (Sinn, Germany), and Corning Inc. (Corning, NY, USA). The used antibodies—mouse monoclonal anti-cytochrome C, mouse monoclonal anti-β-actin, and mouse monoclonal anti-lamin A/C—were from Santa Cruz Biotechnology, Inc. (Santa Cruz, CA, USA). The monoclonal mouse anti-*O*-GlcNAc (RL2) (ab2739), monoclonal mouse anti-BrdU (IIB5) (ab8152), and polyclonal goat anti-mouse IgG H&L FITC (ab6785) antibodies were from Abcam (Cambridge, UK). Rabbit polyclonal anti-*O*-GlcNAcase (OGA) (SAB4200311) and rabbit polyclonal anti-*O-*GlcNAc transferase (OGT) (Ti-14) antibodies were from Sigma-Aldrich (St. Louis, MO, USA). Rabbit monoclonal anti-OGT (G921A), secondary mouse anti-rabbit (7074), and goat anti-mouse (7076) IgG-HRP antibodies were purchased from Cell Signaling Technology, Inc. (Beverly, MA, USA). Mouse monoclonal anti-HaloTag antibodies were from Promega^®^ (Madison, WI, USA). 

### 2.2. DNA Constructs

**Figure 2:**
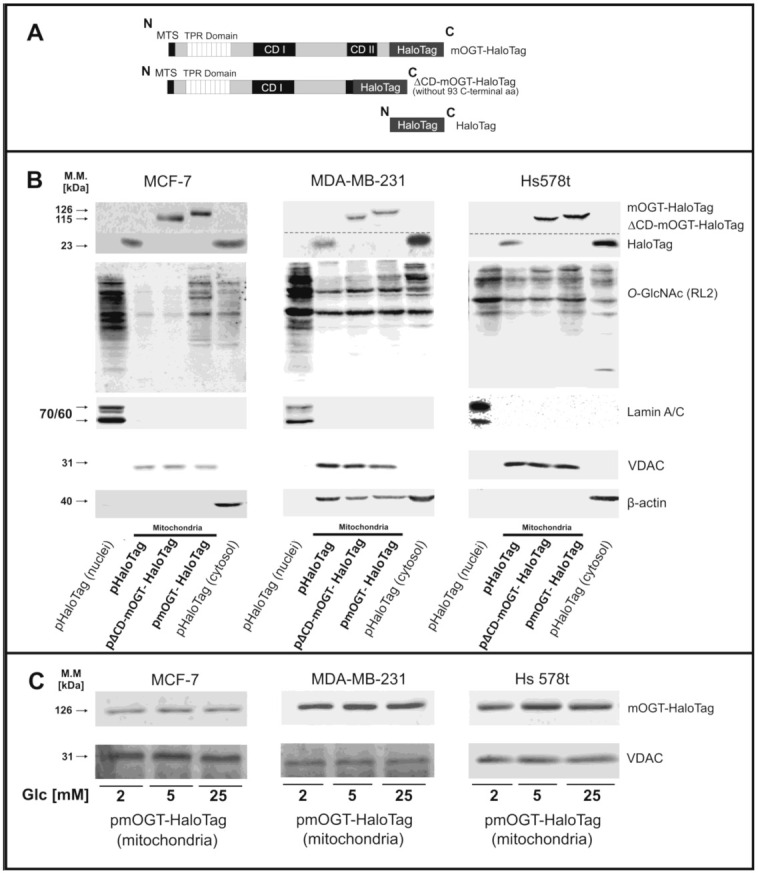
Up-regulated mOGT targets the mitochondria and modifies mitochondrial proteins in breast cancer cells. (**A**) The scheme of mOGT-HaloTag fused protein, its catalytic inactive mutant with truncated CDII domain, and HaloTag used as a control. (**B**) Expression of Halo-tagged proteins and *O*-GlcNAc level in mitochondria-enriched fractions 48 h after plasmid transfection of cells growing in hypoglycemia. Nuclei-enriched fraction and cytoplasm extract were loaded to assess the purity of mitochondria. VDAC, Lamin A/C, and β-actin were used as purity markers for mitochondria, nucleus, and cytoplasm, respectively. (**C**) Expression of mOGT-HaloTag in mitochondria-enriched fraction derived from cells cultured in hypo-, normo-, or hyperglycemia conditions. MTS, mitochondria-targeted sequence; TPR, tetratricopeptide repeats, CD, catalytic domain; Glc, glucose. (The original western blot images are shown in the File S1).

The full-length human mOGT gene reported in GenBank^TM^ (accession number U77413) was synthesized using GeneArt service (Invitrogen^TM^, Carlsbad, CA, USA). The ∆CD-mOGT catalytically inactive mOGT mutant was generated using the sequence encoding mOGT but without the last 93 amino acid region (Figure 2A). 

The codon sequences were normalized by GeneOptimizer^TM^ software (Thermo Fisher Scientific, Waltham, MA, USA) in order to obtain a high yield of mRNAs and proteins from synthetic genes. Each synthesized construct was flanked by SgfI and PmeI sites in pMA vector and subcloned into the pFC27K HaloTag^®^ CMV-neo Flexi^®^ Vector (#G8431, Promega^TM^) following the manufacturer’s instructions and transformed into competent JM109 cells (Promega^TM^, Madison, WI, USA). These CMV-driven constructs produce fusion proteins tagged by the HaloTag protein, which consists of a 33 kDa monomeric protein not expressed in mammalian, plant, or *E. coli* cells. The empty vector that only encodes HaloTag protein was generated by blunt ends ligation of purified product generated after PCR amplification using designed primers and the Physion^TM^ (Thermo Scientific^TM^, Waltham, MA, USA) High-Fidelity DNA Polymerase (Thermo Scientific^TM^). Plasmids were isolated using Extract Me Plasmid Maxi Endotoxin-Free Kit (Blirt^®^, Gdańsk, Poland) and validated by direct DNA sequencing. 

### 2.3. Cell Culture and Treatment

MCF-7, MDA-MB-231, and Hs578t breast cancer cell lines were obtained from the American Type Culture Collection (Manassas, VA, USA). Cells were grown in Dulbecco’s modified Eagle’s medium (DMEM) supplemented with 10% (*v*/*v*) fetal bovine serum (FBS). All cell lines were grown in a humidified atmosphere containing 5% (*v*/*v*) CO_2_ at 37 °C. To assess the impact of glucose on levels of *O*-GlcNAcylation as well as expression of proteins, cells were grown for 72 h in medium containing 2, 5, or 25 mM glucose concentrations, which in blood correspond to hypo-, normo-, and hyperglycemia conditions, respectively. Transfections with vectors encoding mOGT, ∆CD-mOGT, or HaloTag protein alone were performed using Lipofectamine^TM^ 2000 (Invitrogen^TM^, ThermoFisher Scientific, Grand Island, NY, USA) transfection reagent. Cells were seeded on plates at 90% confluence and then transfected for 24 or 48 h with 0.1 μg of plasmid and 0.2 µL Lipofectamine per well (96-well plate) or 2 μg of pDNA and 4 µL of Lipofectamine per well (6-well plate) according to the manufacturer’s instructions. For experiments dedicated to the identification of mOGT’s protein substrates and binding partners by mass spectrometry, cells were co-transfected with plasmids in combination with Silencer^TM^ Pre-designed siRNA targeted ncOGT or with Silencer^TM^ Negative Control No.1 siRNAs (Cat. Nr. AM16704 and AM4611, respectively; Ambion^®^, (Milwaukee, WI, USA). Initially, cells plated on 10 cm dishes at 70% confluence were transfected for 72 h with 100 pmol siRNA in the presence of 10 µL Lipofectamine RNAiMAX (Invitrogen^TM^, ThermoFisher Scientific, Grand Island, NY, USA) following manufacturer’s specifications. After 24 h, cells were co-transfected with 10 μg of plasmid using Lipofectamine 2000 transfection reagent. Cells were then incubated for 48 h. For all experiments, cells were plated in triplicates.

### 2.4. RT-PCR

RNA was isolated from the breast cancer cells using the total RNA isolation kit (A&A Biotechnology, Gdynia, Poland) according to the manufacturer’s instructions. First-strand cDNAs were obtained by the reverse transcription of 1 μg of total RNA using a High-Capacity cDNA Reverse Transcription kit (Applied Biosystems, Foster City, CA, USA); then, qPCR was performed using HOT FIREPol^®^EvaGreen^®^ qPCR Mix Plus (Solis BioDyne, Tartu, Estonia) according to the manufacturer’s instructions. The following sets of primers were employed to measure the expression levels of mOGT and ncOGT and the internal controls HPRT1 or GAPDH by qPCR: mOGT, 5’-tggctggtcagagaaggaataa-3’, and 3’-gacgttggatcggttacgag-5’; ncOGT, 5’-gcaacctagccaatgctctc-3’, and 3’-cagaagggtctcaaacgacg-5; HPRT1 5’-ccctggcgtcgtgattagtg-3’ and 3’-cctgacttgcagaacgagct-5’; GAPDH 5’-cctgcaccaccaactgctta-3’ and 3’-aggtcttgtagtagggacgg-5’. Detailed information concerning the primers is presented in File S2 Supplementary Methods. The reactions were performed in triplicate using Eppendorf RealPlex thermal cycler (Enfield, CT, USA). PCR products were confirmed by melting curves analysis and were tested by electrophoresis with ethydium bromide staining. The abundance of studied genes mRNA in samples was quantified by the ΔC method. Ct (Ct_gene_–Ct_GAPDH_); values were recalculated into relative copy number values (number of copies of gene of interest mRNA per 1000 copies of reference gene mRNA).

### 2.5. Isolation of Mitochondrial, Nucleus, and Cytosol Fractions

Mitochondria were purified from cells using Mitochondria Isolation Kit for Cultured Cells (Thermo Scientific) according to manufacturer’s instructions, with minor modifications. Unless specified otherwise, all steps were carried out at 4 °C. A total of 2 × 10^7^ cells were briefly suspended in Mitochondria Isolation Reagent A and incubated for 2 min on ice. Cells were transferred into a Glass-Teflon homogenizer and disrupted by ~25–30 strokes. The lysis efficiency was determined by visual estimation using a microscope. Subsequently, Mitochondria Isolation Reagent C was added, and then lysed cells were centrifuged at 700× *g* for 10 min to remove cell debris and remaining nuclei. For SDS-PAGE, pellets containing nuclei were purified three times by suspension in Reagent C followed by centrifugation at 700× *g* for 10 min. The lacking nuclei supernatants were also spun again at 700× *g* for 10 min to remove the remaining nuclei. Then, supernatants were transferred into new vials and spun at 3000× *g* for 15 min. The pellets containing mitochondria were resuspended in Mitochondria Isolation Reagent C and centrifuged at 12,000× *g* for 10 min. Mitochondria-enriched pellets were collected and used for subsequent experiments. Cytosol fractions were purified by twice centrifugation at 12000× *g* for 10 min of supernatants collected following mitochondria sedimentation. 

### 2.6. Western Blotting

Proteins were resolved by SDS-PAGE and electroblotted onto Immobilon-P transfer membranes. The blots were incubated with primary antibodies for 2 h at room temperature. After washing three times with Tris-buffered saline (TBS), the blots were incubated for 1 h with horseradish peroxidase-coupled secondary antibodies. Proteins were visualized on X-ray films or by using a CCD camera by an enhanced chemiluminescence method using SuperSignal^TM^ West Pico PLUS Chemiluminescent Substrate (Thermo Scientific^TM^). For loading control, the blots were re-probed with anti-β-actin, anti-cytochrome C, anti-VDAC1, anti-VDAC3, or anti-lamin A/C antibodies following a stripping protocol. 

### 2.7. Determination of Mitochondrial ROS Production

Mitochondrial ROS production was evaluated using MitoSOX assay. Cells were seeded onto 12-well plates and incubated overnight. Cells were treated for 24 or 48 h with plasmid DNA followed by staining with MitoSOX Red mitochondrial superoxide indicator (Molecular Probes, Eugene, OR, USA). After washing of the cells, the specific localization of the dye was confirmed using a fluorescence microscope (Olympus IX70, Melville, NY, USA)) and then the fluorescence intensity of the cells was assessed by flow cytometry (excitation wavelength: 510 nm; emission wavelength: 580 nm; cytometer Becton DickinsonLSR^®^, (Bergen, NJ, USA).

### 2.8. ATP Level Measurement

ATP level was determined by using a Luminescent ATP Detection Assay Kit (ID: ab113849; Abcam^®^, Cambridge, UK) per the manufacturer’s instruction. Briefly, cells were seeded in a 96-well plate (μClear^®^, white, flat bottom; Greiner Bio-One GmbH, Frickenhausen, Germany) one day prior to the treatment. Cells growing in media with different glucose concentrations (low, medium, and high) were transfected with plasmid DNA for 24 or 48 h, and then ATP level was measured. Cell lysates were treated with ATP detection reagent for 10 min at room temperature. The luminescent signal was quantified by a plate reader (SpectraMax^®^ PARADIGM^®^ Multimode Microplate Plate Reader; Molecular Devices, CA, USA). Cellular ATP levels were calculated from a standard curve (μM).

### 2.9. Mitochondrial Membrane Potential Assay

Mitochondrial membrane potential was assessed in cells cultured in 96-well plate (μClear®, black, flat bottom; Greiner Bio-One GmbH, Frickenhausen, Germany). Cells were stained with cell-permeable potentiometric fluorescent dye JC-1 (5,5’,6,6’-tetrachloro-1,1’,3,3’-tetraethylbenzimidazolycarbocyanine iodide) as described previously [22]. Cells were labeled with JC-1 at a concentration of 2 μM and incubated at 37 °C for 30 min before being washed once with pre-warmed (37 °C) PBS. For the positive control, cells were incubated with 50 μM final concentration of CCCP before staining. The fluorescence was measured on a Bio-Tek Synergy HT Microplate Reader (Bio-Tek Instruments, Winooski, VT, USA), with filter pairs of 485 nm/538 nm and 530 nm/590 nm. Results were shown as a ratio of fluorescence, measured at 530 nm/590 nm to that measured at 485 nm/538 nm (aggregates to monomer fluorescence).

### 2.10. Cell Imaging

For experiments with RNA interference, cells were plated at a density of 1.5 × 10^4^ cells/well for 24 h and then treated with siRNA duplexes for 72 h, as described in the cell culture and treatment section. Following transfection, cells were fixed in 4% (*m*/*v*) paraformaldehyde, washed in PBS, quenched in 100 mM glycine, washed in PBS, and permeabilized in PBS containing 0.5% (*v*/*v*) Triton X-100 for 20 min. Then, cells were blocked in blocking buffer (2% (*v*/*v*) Fetal Calf Serum (FCS)-2% (*m*/*v*) Bovine Serum Albumin (BSA)-0.2% (*m*/*v*) gelatin) for 1 h at room temperature. Cells were immunostained for OGT (Ti-14, Sigma-Aldrich) at a 1:100 dilution in blocking solution overnight at 4 °C. After washing in PBS, cells were incubated with the Alexa Fluor 647-conjugated goat anti-rabbit IgG antibodies (1:600; Invitrogen) for 1 h at room temperature. Cells were then washed with PBS and counterstained with DAPI (50 μg/mL, Molecular Probes) to visualize nuclei. Immunolabeled cells were imaged at 25 °C at a magnification of 63× by using a Leica TCS SP8 system.

### 2.11. Enrichment of Mitochondrial O-GlcNAcylated Proteins and Isolation of mOGT-Interacting Protein Partners by Immobilization of HaloTag^®^ Fusion Proteins

Immunoprecipitation was performed using RL2 antibody on mitochondria-enriched fractions from cells co-transfected with plasmids (empty backbone; ΔCD-mOGT-HaloTag; mOGT-HaloTag) and ncOGT siRNA. Immunoprecipitates were carried out using Pierce^®^ Crosslink Immunoprecipitation Kit (Thermo Scientific, Rockford, IL, USA) according to the manufacturer’s instruction. Mitochondria-enriched samples were incubated for 30 min in a lysis buffer (50 mM Tris-HCl, 150 mM NaCl, 1% (*v*/*v*) NP-40, 0.5% (*m*/*v*) sodium deoxycholate, 0.1% (*m*/*v*) SDS, pH 7.4, supplemented with Halt^TM^ protease Inhibitor Cocktail (Thermo Scientific, Rockford, IL, USA) on ice. The lysates were mixed at a volume ratio 1:9 with Pierce^®^ IP Lysis buffer (25 mM Tris-HCl, 150 mM NaCl, 1 mM EDTA, 1% (*v*/*v*) NP-40, 5% (*v*/*v*) glycerol, pH 7.4, supplemented with Halt^TM^ Protease Inhibitor Cocktail (Thermo Scientific, Rockford, IL, USA) and then centrifuged at 13,000× *g* for 10 min. Supernatants (0.5–0.7 mg of protein) were pre-cleared using control agarose resin. Immunoprecipitation experiments were carried out by incubating pre-cleared lysates with antibody-crosslinked resin at 4 °C with gentle rolling overnight. After four washes, the beads were incubated with Laemmli sample buffer containing 5% (*v*/*v*) 2-mercaptoethanol and heated at 95 °C for 5 min. 

Isolation of protein partners interacting with mOGT-HaloTag, ΔCD-mOGT-HaloTag fusion proteins, or HaloTag protein only were done by pull-down assay using HaloLink^TM^ Resin (Promega^TM^, Madison, WI, USA) according to manufacturer’s instructions. For this method, HaloTag binding ligand was covalently attached to the surface of Sepharose beads via a carbamide linkage. Covalent binding of HaloTag^®^ fusion proteins to the HaloLink^TM^ Resin allows extensive washing of the bait-prey complexes without the risk of dissociation from the resin. About 500 μg of proteins derived from mitochondria-enriched lysates of cells transfected with plasmid DNA was added to 150 μL equilibrated HaloLink^TM^Resin resuspended in binding buffer (100 mM Tris-HCl, 150 mM NaCl, 0.05 % (*v*/*v*) IGEPAL^®^ CA-630, pH 7.6) and then incubated by mixing overnight on a tube rotator at 4°C. The precipitates were washed four times with 1 mL of buffer containing 100 mM Tris-HCl, −150 mM NaCl, 1 mg/mL BSA 0.5%, IGEPAL^®^ CA-630 pH 7.6) followed by centrifugation at 800× *g* for 2 min. Finally, proteins were eluted by heating at 95 °C with a Laemmli sample loading buffer (2×).

### 2.12. Identification of mOGT’s Protein Partners and Protein Substrates by Mass Spectrometry

#### 2.12.1. Chemicals

Pierce^®^ LTQ ESI positive ion calibration solution and Pierce™C18 tips were purchased from Thermo Fisher Scientific (Rockford, IL, USA). LC–MS-grade acetonitrile, HPLC-grade formic acid and trifluoroacetic acid, MS Qual/Quant QC Mix, were obtained from Sigma-Aldrich (St. Louis, MO, USA). Water was purified by a Milli-Q water purification system (Millipore Corp., Bedford, MA, USA).

#### 2.12.2. Sample Preparation Prior to LC–MS/MS 

The eluted proteins were resolved by SDS-PAGE, and gels were stained using brilliant blue staining. Each lane was cut into small slices and placed into 0.65 mL siliconized tubes. The slices were washed thrice in ~100 μL of 25 mM NH_4_HCO_3_/50% (*v*/*v*) ACN by vortexing for 10 min followed by complete drying of the gel pieces using a Speed Vac centrifuge (Eppendorf). The dried gels were incubated with 10 mM DTT in 25 mM NH_4_HCO_3_ at 56 °C for 1 h. Supernatants were removed, and the gel pieces were covered by 55 mM iodoacetamide in 25 mM NH_4_HCO_3_ and were placed in the dark for 45 min at room temperature. After this step, gels were washed by vortexing for 10 min with ~100 μL NH_4_HCO_3_, then shaken twice with ~100 μL of 25 mM NH_4_NCO_3_ in 50% (*v*/*v*) ACN for 5 min. Dehydrated gels were centrifuged to complete dryness using a Speed Vac centrifuge (Eppendorf). Next, the gel pieces were rehydrated at 4 °C with 25 μL of Trypsin Gold (Promega, Madison, WI, USA) solution (12.5 ng/μL trypsin in 25 mM NH_4_CO_3_, freshly diluted). As needed, a solution of 25 mM NH_4_HCO_3_ was added to cover the slices. Digestion was performed overnight at 37 °C. The digest solution was transferred into a clean 0.5 mL siliconized tube. The gel pieces were mixed twice with 50 μL of 50% (*v*/*v*) ACN/5% (*v*/*v*) formic acid. The digests were dried using a Speed Vac centrifuge, then re-suspended in 0.1% (*v*/*v*) trifluoroacetic acid (TFA) in water and purified by the Pierce™ C18 tips according to the manual instructions. Briefly, C18 tips were activated with 100 µL acetonitrile (e.g., 8–10 up–down cycles when attached to a 10 µL pipette) and stabilized by 50% (*v*/*v*) acetonitrile and 0.1% (*v*/*v*) TFA in water. Next, 10 µL of sample was loaded into the Pierce™ C18 tip (with 10 up–down cycles of the pipette). Then, the trapped peptide sample was washed with 100 µL 0.1% (*v*/*v*) TFA. Finally, the sample was eluted from the Pierce™ C18 tip using 20 µL of 0.1% (*v*/*v*) formic acid in a 95% (*v*/*v*) acetonitrile into a vial for LC–MS/MS analysis.

#### 2.12.3. LC–MS/MS Analysis

LC–MS/MS analysis was performed using a Transcend™ TLX-2 multiplexed LC system equipped with Q-Exactive Orbitrap mass spectrometer (Thermo Scientific, Hudson, New Hampshire, USA) using a heated electrospray ionization (ESI) interface (HESI–II) according to the method described by Kockmann et al. [23], Geiger et al. [24], and Velloso et al. [25], with some modifications. Samples were separated using an aC18 Acclaim PepMap^TM^ 100 column (1.0 × 150 mm, 3 μm particle size, nanoViper, Thermo Fisher Scientific, PA, USA) thermostated at 25 °C. The mobile phases were eluent A, FA/water (0.1/99.9, *v*/*v*) and eluent B, FA/acetonitrile (0.1/99.9, *v*/*v*). The flow rate was 75 µL/min, and the gradient was as follows: 0–0.25 min, 2% B; 0.25–54 min, 2–35% B; 54–75 min, 35–2% B; 2% B was maintained for another 15 min for column re-equilibration. The sample injection volume was 10 μL. The Q-Exactive Orbitrap mass spectrometer was operated in a positive ionization mode with full MS and a subsequent all-ion fragmentation (AIF) mode. The accuracy and mass calibration were done according to the manufacturer’s recommendations using a mixture of standards in the mass range of *m*/*z* 138.06619–1779.96528. The capillary temperature was adjusted to 250 °C and aux gas heater temperature to 100 °C. The electrospray capillary voltage and S-lens radio frequency (RF) level were set at 2.5 kV and 50 V, respectively. Nitrogen was used as both sheath gas and auxiliary gas at a flow of 5 and 1 (arbitrary units), respectively. The acquisition method consisted of two scan events, full MS-SIM and AIF. The full MS-SIM scan spectra were acquired over an *m*/*z* range of 350–2000, with the mass resolution of 17,000 full-width at half maximum (FWHM) at *m*/*z* = 200. Automatic gain control (AGC) target (the number of ions to fill C-Trap) was set at 3.0e^6^ with a maximum injection time (IT) of 50 ms. The second scan event was collision-induced dissociation C-trap (CID) with normalized collision energy (NCE) of 25 V, acquired over an *m*/*z* range of 350–2000. In the AIF scan, the mass resolution was 70,000 FWHM at 100 *m*/*z* with AGC target at 2 × 10^5^ maximum IT 100 ms. Instrument control, data acquisition, and evaluation were done with the Q-Exactive Tune 2.1, Aria 1.3.6, and Thermo Xcalibur 2.2 software, respectively.

#### 2.12.4. Analysis of Proteomic Data

Raw MS/MS data were analyzed using Proteome Discoverer 2.4.0.305 (Thermo Fisher Scientific). MS/MS spectra were searched against a human FASTA-formatted database (SwissProt, v2017–10-25 with taxonomy Homo sapiens, 42,252 sequences) using the SEQUEST HT algorithm. All database search was performed using a precursor mass tolerance of ±20 ppm and a fragment ion mass tolerance of ±0.6 Da. Enzyme specificity was selected to trypsin. Minimal peptide length was set to six amino acids with a maximum missed cleavages value of 2. Database searches were performed with carbamidomethylation on cysteine as static modification and oxidation on methionine and acetylation of protein N-terminal as possible modifications. For peptide and protein identification, the false discovery rate (FDR) with a target-decoy strategy was set to 0.01.

#### 2.12.5. Statistical Analysis

Data presented are the mean ± standard error of the mean (SEM). Statistical evaluation was performed using STATISTICA data analysis software (ver. 13; StatSoft Inc., Tulsa, OK, USA). The Student’s paired t-test was used to compare the differences between treated and untreated cells. A *p*-value of <0.05 was considered significant.

## 3. Results

### 3.1. OGT Expression and O-GlcNAc Level Are Glucose-Dependent in Mitochondria of Breast Cancer Cells

Previous observations indicate that global cellular *O*-GlcNAc level and expression of enzymes associated with this process are strongly regulated by glucose availability [15,26,27]. Since the recent data concerning the role of ncOGT and mOGT in mitochondrial protein *O*-GlcNAcylation are ambiguous, we first verified the expression of both splice variants in breast cancer cells cultured in different glucose conditions. To separately detect sequences encoding mOGT or ncOGT, we designed specific sets of primers. Using a RT-qPCR method, along with standardization against two housekeeping genes, we observed that the level of mRNA encoding both mOGT and ncOGT in breast cancer cells were significantly decreased in high glucose concentration (Figure 1B). The strongest impact of glucose concentration on *OGT* gene expression level was monitored for Hs578t cells. Each cell lines expressed mOGT mRNA at levels approximately 5–10 fold lower compared to those encoding ncOGT. However, mRNA levels encoding both isoforms seem to be affected in the same way by glucose content.

Mitochondrial localization of mOGT was previously detected by Love et al. [28] in HeLa cells by using Western blot, although no data supports this observation in other human cell lines [19]. Since glucose impacts on OGT mRNA levels, we assessed mOGT and ncOGT expression at the protein level in mitochondria-enriched fraction and cytoplasmic fraction of cells grown for 72 h in low (2 mM) and high (25 mM) glucose concentrations (Figure 1C). As reported earlier [16,28], we detected a ~103 kDa immunoreactive band in the mitochondria fraction, whereas a ~117 kDa band corresponding to ncOGT was only observed in the cytosolic fraction (Figure 1C). The expression of the ~103 kDa isoform was inversely correlated to glucose concentration; on the other hand, the cytosolic ncOGT expression seems to be only slightly down-regulated by high glucose in MCF-7 and MDA-MB-231 cells, with no difference observed in Hs578t cells. Interestingly, in opposition to the glucose-dependent decrease in mOGT expression, the level of *O*-GlcNAcylated mitochondrial proteins was higher in the high glucose condition when compared to the low glucose concentration. We then monitored the OGA expression in the two fractions, the localization of this enzyme in mitochondria having been recently confirmed [18]. Even though we detected OGA both in mitochondrial and cytoplasmic fractions, the level of this enzyme was unchanged under the different glucose conditions. Together, these observations suggest that glucose availability regulates OGT level in mitochondria, likely by control of gene expression. The increased *O*-GlcNAc level in mitochondria despite the decreased level of OGT may be due to changes in OGA and OGT catalytic activity or increased UDP-GlcNAc level.

### 3.2. mOGT Up-Regulation in Breast Cancer Cells Increases Mitochondrial O-GlcNAc Level

To better understand the role of mOGT in the regulation of mitochondria activity and bioenergetics and to identify mOGT protein partners in breast cancer cells, we designed plasmids that encode full-length mOGT and a mOGT mutant lacking the second catalytic domain (CDII). These constructs were cloned into commercial vectors that enable their expression in breast cancer cells as fusion proteins with HaloTag located at the C-terminus (mOGT-HaloTag and ∆CD-mOGT-HaloTag; for the full-length and the catalytic dead isoforms, respectively). Initially, we tried to generate stable cell lines by exerting a selective pressure with G418. However, similarly to another group [29], we could not generate stable cell lines overexpressing active mOGT, this was probably due to mOGT cytotoxic effect. Therefore, our study was conducted using transient expression of mOGT-HaloTag or ∆CD-mOGT-HaloTag proteins *versus* only HaloTag protein as a control (Figure 2A). As shown in the Figure 2B, mOGT-HaloTag, ∆CD-mOGT-HaloTag proteins, and HaloTag alone were targeted to the mitochondria-enriched fraction. As expected, in cells transfected with pmOGT-HaloTag, the level of *O*-GlcNAcylated proteins in mitochondria was increased in contrast with cells transfected with *p*-∆CD-mOGT-HaloTag, confirming the catalytic activity of mOGT-HaloTag in mitochondria. The results of densitometric analysis are shown in Figure S1. While levels of endogenous mOGT were dependent upon glucose concentrations (Figure 1C), the expression of mOGT-HaloTag in mitochondria-enriched fractions is independent of glucose availability (Figure 2C). Therefore, these findings suggest that mOGT expression in mitochondria is more likely dependent upon gene expression rather than proteostasis. 

### 3.3. Increased mOGT Expression Affects Mitochondrial Homeostasis and Cellular Energy Metabolism

**Figure 3:**
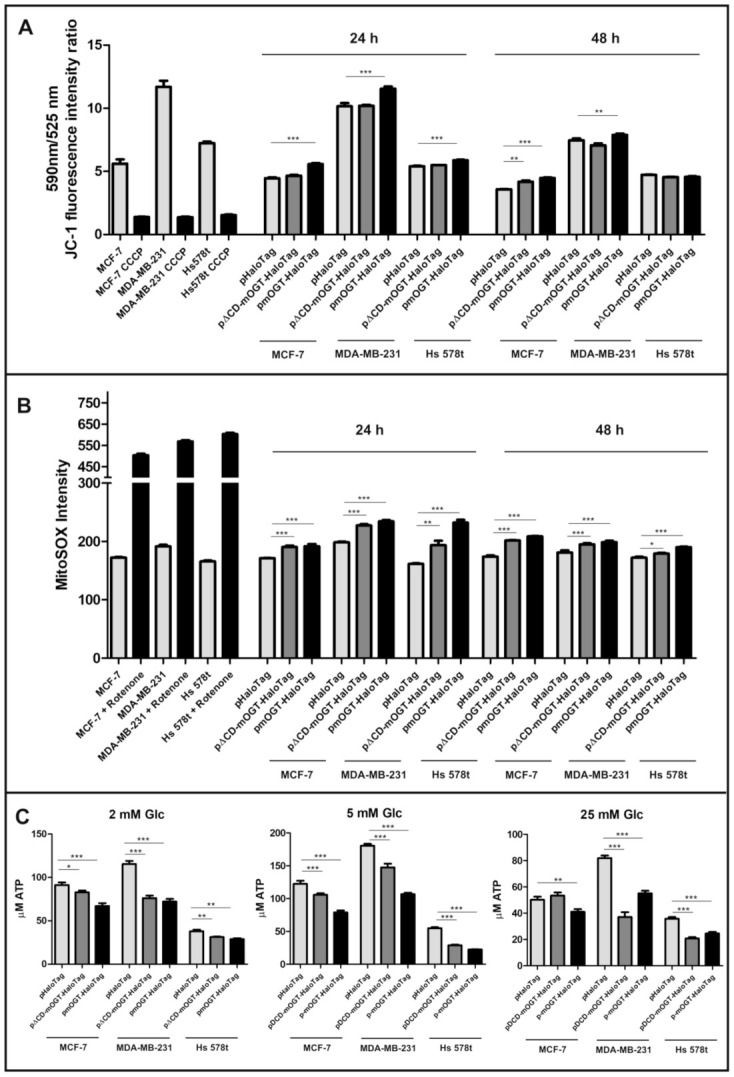
Elevated mOGT level impairs mitochondria homeostasis and ATP production. (**A**) Determination of mitochondrial transmembrane potential 24 or 48 h after transient transfection of cells with plasmid DNA. The 590/525 nm fluorescence intensity ratio of JC-1 dye is presented as a mean +/− SD. (**B**) Mitochondrial ROS level in transfected cells assessed by using MitoSOX Red mitochondrial superoxide indicator. The bars are shown as a median +/− SD of fluorescence intensity of cells determined by flow cytometry. (**C**) Intracellular ATP contents in cells treated for 48 h with pHaloTag (control), pΔCDmOGT-HaloTag, or pmOGT-HaloTag were determined using a Luminescent ATP Detection Assay Kit. (Data represent the average of at least 3 independent experiments performed in tetraplicate. * indicates significance *p* < 0.05; ** *p* < 0.01; *** *p* < 0.001. CCCP is a chemical agent which allows for complete dissipation of the proton gradient across the mitochondrial inner membrane.

According to published data, the increase in *O*-GlcNAcylation of mitochondrial proteins contributes to impaired mitochondrial function. More precisely, *O*-GlcNAcylation regulates oxidative phosphorylation at multiple sites of the respiratory chain contributing to alterations of the mitochondrial membrane potential and ATP production rates [15,16,17]. To find out whether mOGT expression and its catalytic activity influence the mitochondrial metabolic state of breast cancer cells, the OGT expression in cells grown in normal glucose conditions was up-regulated by transfection with plasmids encoding either mOGT or catalytic dead version versus the vector only encoding HaloTag as a control. Firstly, we determined the impact of increased-mOGT expression on the mitochondrial membrane potential (ΔΨm), which is considered an index of mitochondrial condition. The transmembrane potential was assessed by staining cells with JC-1, a potentiometric fluorescent dye that accumulates in energized mitochondria. To confirm the sensitivity of the JC-1 dye to the detection of changes in membrane potential, the CCCP control was used. Cells transfected with pmOGT-HaloTag showed a significant increase in mean JC-1 fluorescent shift ratio (in a range from 24% for MCF-7 to 8% for Hs578t cells) compared with the control (pHaloTag transfected) cells (Figure 3A). The mitochondrial membrane potential is the main bioenergetic parameter that controls respiratory rate. Respiration of mitochondria is regulated by both the membrane potential as well as by ATP, which directly inhibits cytochrome C oxidase in a ΔΨm-independent way. To determine whether changes in the respiration rate were associated with mitochondrial ROS production, we stained the cells transfected with DNA vectors with the red fluorescent cell-permeable superoxide indicator MitoSOX. We observed that in all cell lines, the increase in the full-length or catalytically inactive mOGT expression led to a significant increase in mitochondrial superoxide levels (Figure 3B). In cells up-regulating mOGT, the marked increase in ROS levels was accompanied by an increase in mitochondrial membrane potential. The increase in mitochondrial membrane potential and the generation of intramitochondrial ROS are the common features of the early stage of apoptosis [30].

In order to determine the influence of mOGT on the energy state of breast cancer cells, we assayed the intracellular levels of ATP in cells transfected with pmOGT-HaloTag or pCD-mOGT-HaloTag *versus* pHaloTag and grown under different glucose concentrations. For the three breast cancer cell lines, expression of either mOGT-HaloTag or pCDmOGT-HaloTag caused a decrease in intracellular ATP levels in comparison to pHaloTag treated cells (Figure 3C). The most significant differences in ATP levels were observed for MDA-MB-231 cells. A decrease in ATP levels in cells was observed independently of the concentration of glucose.

Together, these observations suggest that the up-regulation of mOGT limits the mitochondrial ATP production and increases the generation of reactive oxygen species, which are probably responsible for the toxic effects of mOGT. 

### 3.4. mOGT Interacts and O-GlcNAcylates Multiple Sets of Mitochondrial Proteins

**Figure 4:**
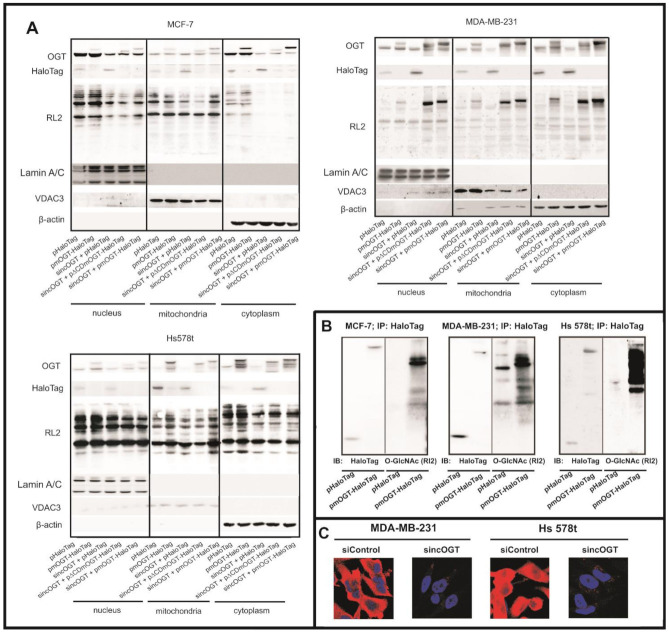
Isolation of mOGT candidate interacting proteins and modified substrates by proteomics approaches. (**A**) Representative Western blots present the effect of ncOGT knock-down and mOGT over-expression on the OGT and *O*-GlcNAc modified protein levels in nuclei- and mitochondria-enriched fractions and cytoplasm extracts from breast cancer cells. Blots were re-probed with Lamin A/C, VDAC3, and β-actin antibodies to verify the purity of isolated fractions. The mitochondrial lysates present herein were used for isolation of mOGT interactors or proteins modified by mOGT. (**B**) The confocal images of paraformaldehyde-fixed cells immunolabelled for OGT (red) counterstained with DAPI (blue) showing efficiency of ncOGT RNA interference. (**C**) mOGT-interacting proteins isolated from mitochondria-enriched lysates by using HaloLink Resin were probed with HaloTag and *O*-GlcNAc antibodies. (The original western blot images are shown in the File S1).

**Figure 5:**
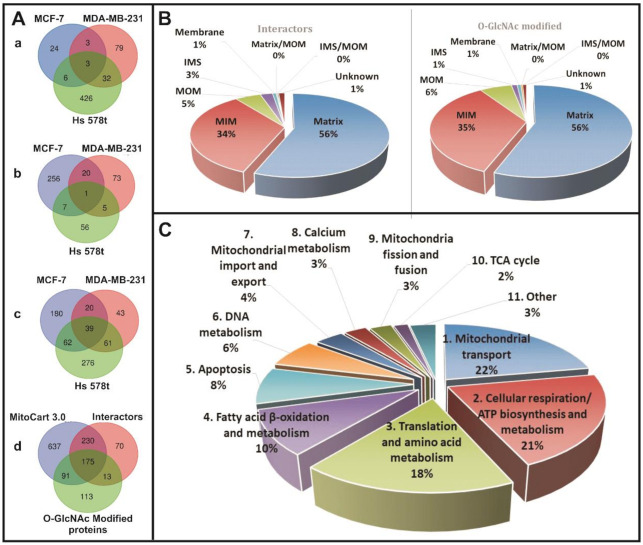
Proteomic analysis of isolated mOGT interactors and *O*-GlcNAc-modified candidates from mitochondria-enriched lysates of three analyzed cell lines. (**A**) Venn diagrams showing a, the total number of identified mOGT mitochondrial interactors; b, the total number of identified mOGT modified mitochondrial candidates; c, the total number of all identified mOGT protein partners and substrates; d, the interactors and *O*-GlcNAcylated candidates common with mitochondrial proteins according to MitoCart 3.0 datasets (Broad Institute). (**B**) The graph presents submitochondrial localization of identified interactors and *O*-GlcNAc modified candidates according to MitoCart 3.0 datasets. (**C**) The graph presents identified mitochondrial mOGT candidate proteins distributed into biological functional classes. MOM, mitochondrial outer membrane; IMS, intermembrane space; MIM, mitochondria inner membrane.

According to our results, mOGT has its own catalytic activity and is able to *O*-GlcNAcylate a myriad of mitochondrial proteins. To identify proteins interacting with the mOGT, we employed a HaloLink^®^ Resin to co-purify the mOGT-HaloTag fusion protein with its mitochondrial interacting partners (HaloTag alone was used as negative control; see Methods). Transient transfections and pull-down efficiency were verified by Western blotting (Figure 4A,B). The enriched proteins were identified by nano-LC–MS/MS as described in the Methods. The candidate proteins interacting with mOGT were selected by a comparative analysis of the mOGT-HaloTag versus HaloTag protein partners. We identified many candidates interacting specifically with mOGT in breast cancer cells (Tables S1 and S6). Some of them were identified only in one cancer cell type, but 46 proteins were found in at least two of the three analyzed cell lines (Figure 5A,a). In the second approach, we intended to find out which mitochondrial proteins are *O*-GlcNAcylated by mOGT. Since *O*-GlcNAcylation of mitochondrial proteins can involve both mOGT and ncOGT, we used the RNA interference method to specifically knock-down ncOGT and increase the expression of mOGT by transient transfection. The sincOGT duplexes matched to the ncOGT mRNA sequence encoded by exon 2 of *OGT* gene; therefore, they specifically silenced only the long OGT isoform. In addition, the sequence of the mOGT-expressing vector was resistant to RNA interference. The efficiency of siRNA duplexes was checked by staining cells with anti-OGT antibodies and by visualization of the cells by confocal microscopy (Figure 4C). The efficiency of siRNA and pmOGT-HaloTag co-transfection on OGT and *O*-GlcNAcylation levels in the nuclear, mitochondrial, and cytoplasmic fractions of the three breast cancer cell lines was verified by Western blot (Figure 4A). Of particular interest, we noted that ncOGT silencing had no significant impact on OGT expression and *O*-GlcNAcylation levels in the mitochondrial fraction. On the other hand, as expected, a marked decrease in OGT expression and *O*-GlcNAcylation level was observed in the cytoplasmic and nuclear fractions. These observations suggest that ncOGT does not play a major role in *O*-GlcNAcylation of mitochondrial proteins in breast cancer cells. 

Isolation of *O*-GlcNAcylated proteins from the mitochondrial-enriched fractions of cells previously transfected with sincOGT/p∆CD-mOGT-HaloTag, sincOGT/pmOGT-HaloTag or sincOGT/pHaloTag as a negative control was performed using the well characterized anti-*O*-GlcNAc antibody RL2 and the Pierce^®^ Crosslink Immunoprecipitation Kit. Isolated proteins from *O*-GlcNAc-enriched probes were identified by nano-LC–MS/MS (see Methods). A previous study regarding the structure of human OGT in complex with a peptide substrate reported that the UDP moiety of UDP-GlcNAc binds in a pocket in the C-Cat domain (CDII) near the interface with the N-cat domain (CDI) containing two helices, which form an essential part of the enzyme active site [31]. In our study, the cells transfected with p∆CD-mOGT-HaloTag had slightly lower levels of *O*-GlcNAc modified proteins in mitochondria than the cells expressing HaloTag. We assume that this observation is associated with competition for binding to protein substrates between catalytic inactive ∆CD-mOGT-HaloTag and endogenous mOGT. Therefore, the mOGT-mediated *O*-GlcNAcylation of protein candidates was selected by a comparative analysis between samples that were co-transfected with sincOGT/pmOGT-HaloTag to those co-transfected with sincOGT/p∆CD-mOGT-HaloTag. 

In the second step, all proteins identified in mitochondria of cells expressing only HaloTag were excluded. In this way, we identified a set of 33 *O*-GlcNAcylated candidates potentially modified by mOGT in at least two of the three tested cell lines (Figure 5A,b; Table S2). Moreover, some of these *O*-GlcNAcylated proteins were also identified as interacting partners of mOGT by using HaloLink Resin (Figure 5a,c; Table S3). We compared the identified in our study mOGT interactors and substrates with known mitochondrial proteins according to MitoCart 3.0 datasets (Broad Institute). We found out that 23% (266/1136) of mitochondrial proteins are potentially *O*-GlcNAcylated (Figure 5A,d) and over 35% of them may be interactors with mOGT. Most of these *O*-GlcNAcylated protein candidates are localized in the mitochondrial matrix and inner mitochondrial membrane (Figure 5B) and participate in mitochondrial transport, mitochondrial respiration, translation, fatty acid metabolism, apoptosis, and mtDNA processes (Figure 5C), making mOGT-managed mitochondrial *O*-GlcNAcylation a pivotal process affecting mitochondrial homeostasis. To narrow the list of mOGT interactors and substrates, we prepared a list of 107 proteins that fulfilled the following criteria: (1) were present in at least two of three cell lines, (2) were identified as mOGT interactors, and (3) were identified as *O*-GlcNAcylated proteins (Table S4). Of these 107 proteins, 53 were earlier identified as *O*-GlcNAc modified according to the Human *O*-GlcNAc database [32].

### 3.5. mOGT May Modify Some Nuclear Proteins

The study by Love and collaborators [28] showed that mOGT occurs mainly in the mitochondria thanks to the presence of a mitochondria-targeted sequence in the protein structure. Surprisingly, in our study, we found a set of nuclear proteins as potential mOGT partners or protein substrates. This was not due to contamination of mitochondrial fraction, because we did not detect lamin A/C in our mitochondria-enriched lysates.

The nuclear mOGT’s putative substrates enriched by using both HaloLink Resin and anti-*O*-GlcNAc antibodies (RL2) are presented in Supplemental Table S5. The common candidates with nuclear localization were as follows: Isoform 4 of Apoptotic chromatin condensation inducer in the nucleus, Isoform AML-1L of Runt-related transcription factor 1, Isoform 10 of Transcriptional repressor CTCFL, NK1 transcription factor-related protein 2, and Isoform 2 of Chromodomain-helicase-DNA-binding protein 1.

## 4. Discussion

It has long been known that modification of proteins by *O*-GlcNAcylation controls target protein functions at many levels including subcellular trafficking, stabilization, complex formation, or enzymatic activity, and subsequently modulating metabolic and signaling networks [6]. *O*-GlcNAcylation level is tightly dependent upon HBP flux, which is supplied by diverse nutrients sources such as sugars, fatty acids, and amino acids. Thus, the *O*-GlcNAcylation level may be considered a cellular nutrient sensor [6,33]. Enhanced glucose flux through HBP results in increased *O*-GlcNAcylation. Therefore, since cancer cells metabolism requires accelerated glucose uptake and utilization, this results in an elevated rate of glycolysis and pentose phosphate pathway as well as *O*-GlcNAcylation processes [34]. Thereby, *O*-GlcNAcylation plays a pivotal role in cancer cell signaling and metabolism reprogramming. Champattanachai et al. [12], by using two-dimensional *O*-GlcNAc immunoblotting and LC–MS/MS compared *O*-GlcNAcylation of proteins in breast cancer tissue and normal tissue. They identified 29 proteins with *O*-GlcNAcylation differences, with seven being uniquely *O*-GlcNAcylated in breast cancer. Of these identified proteins, some were related to the Warburg effect, including metabolic enzymes, proteins involved in stress responses, and biosynthesis [12]. Until now, the research on links between *O*-GlcNAcylation and cancer biology has been more heavily concentrated on nuclear and cytoplasmic processes, whereas the reports related to the role of this modification in mitochondria are very limited. Moreover, the role of mOGT in *O*-GlcNAcylation of mitochondrial proteins remains insufficiently understood. To date, the mOGT expression has been reported only in the HeLa cell line [16,28]. In a recent study performed by Trapannone and collaborators [19] on the cell lines IL1R, HEK 293, Jurkat, SH-SY5Y, RAW, U2OS, and A549, surprisingly, mOGT protein was not detected. The authors concluded that this particular OGT isoform may be expressed only transiently, under specific conditions, and ncOGT is sufficient for *O*-GlcNAcylation of mitochondrial proteins. Therefore, as a starting point of this work, we verified mOGT expression in three breast cancer cell lines. For each cell line, the mRNA level encoding mOGT was 5–10 fold lower than ncOGT mRNA, and both of them were reduced upon increase in glucose availability. The glucose-dependent mRNA expression of mOGT and ncOGT was correlated with their protein levels in mitochondria and cytoplasm, respectively. Our results showed that, in breast cancer cells grown in different glucose conditions, mOGT level seems to correlate with expression of ncOGT. Thus, we suppose that mOGT is rather produced as a splice variant of OGT mRNA than driven by an independent promoter of the *OGT* gene. Our study is the first to report on the glucose-dependent expression of mOGT, probably through the effect of glucose on the regulation of *OGT* transcription. In turn, the level of *O*-GlcNAc-modified proteins in mitochondria was closely related to the availability of glucose and inversely correlated with mitochondrial OGT level. These observations are in agreement with previously published data, which showed that cardiac myocytes exposed to high glucose levels induced increased *O*-GlcNAcylation of mitochondrial proteins [15]. According to the literature, most mitochondrial proteins are synthesized in the cytoplasm, and they might be modified by ncOGT before targeting the mitochondria [19]. Thus, the loss of mOGT upon increased glucose levels might be compensated by ncOGT. Moreover, OGA activity can be lower, increasing the occupancy rate of *O*-GlcNAc on mitochondrial proteins. Nevertheless, in our study, the specific down-regulation of ncOGT had no significant impact on mitochondrial *O*-GlcNAc-modified protein level; thus, mOGT isoform may play a pivotal role in *O*-GlcNAcylation processes inside mitochondria and could intervene in reprogramming of mitochondria metabolism in breast cancer cells. 

Actually, increasing evidence points to a critical role for *O*-GlcNAcylation in regulating mitochondrial function and cellular bioenergetics. To explore how increased mOGT expression and *O*-GlcNAcylation affects mitochondria and cellular energy metabolism, we designed and used a plasmid encoding the catalytically active fused mOGT protein and the catalytically inactive mutant displaying a deletion of the second catalytic domain. Our results showed that the increased mOGT expression caused a decrease in intracellular ATP level and altered glycolytic activity in comparison to control cells (Figure 3 and Figure S2). A similar effect associated with a decrease in glycolytic rate and oxygen consumption has been recently observed in SH-SY5Y and NT2 cells with elevated total and mitochondrial *O*-GlcNAcylation following treatment with TMG, a potent OGA inhibitor [35]. However, in a previous study, the authors noted that a decreased mitochondrial *O*-GlcNAcylation in SH-SY5Y cells by up-regulation of OGA expression also caused a significant decrease of oxygen consumption, glycolytic rate, and production of ATP [27]. Therefore, it seems that down-regulation and up-regulation of *O*-GlcNAc have a deleterious effect on mitochondrial function and energy metabolism. Interestingly, another study concerning the role of mitochondrial OGT isoform showed that a reduction of endogenous mOGT by siRNA in HeLa cells is associated with an increase in mitochondrial respiration; however, no significant differences in glycolytic rates were detected [16]. 

The first report regarding mOGT function indicated that mOGT overexpression in INS-1 cells triggers cell cytotoxicity and apoptosis [29]. Up-regulation of mOGT in breast cancer cells increases the mitochondrial membrane potential and the generation of intramitochondrial ROS, often observed in the early stage of apoptosis [30,36]. The same impact of mOGT on mitochondria membrane potential was also described in HeLa cells [16]. Our results argue that the toxic effect of mOGT is more likely related to inhibition of ATP synthesis in mitochondria and ROS production rather than calcium influx to mitochondria (Figure 3 and Figure S3). Similarly, in cardiac myocytes, increased mitochondrial *O*-GlcNAcylation induced by high glucose exposure was associated with impaired activity of complexes I, III, and IV of the respiratory chain in addition to lower mitochondrial calcium and cellular ATP content [15]. As expected, up-regulated mOGT in breast cancer cells had a toxic effect and led to the loss of cell viability independently of the concentration of glucose (Figure S4).

**Figure 6:**
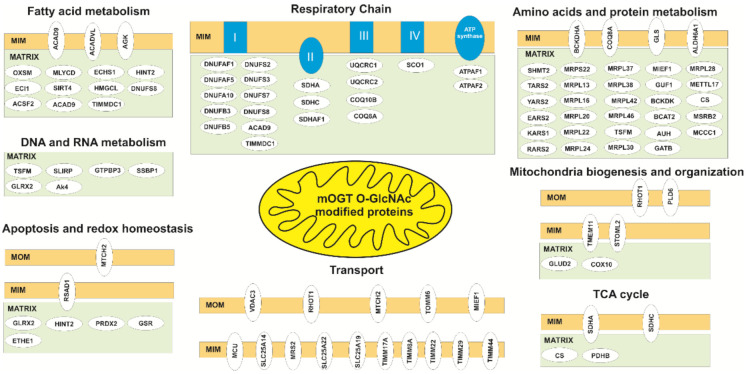
mOGT mediates *O*-GlcNAcylation of proteins involved in many processes central to mitochondria function. The circles indicate identified proteins characterized in Table S4 (Gold List). MON, mitochondria outer membrane; IMS, intermitochondria space, MIM, mitochondria inner membrane.

To shed more light on mOGT significance for mitochondrial activity, we took a proteomics-based approach to identify mOGT-interacting partners and mOGT-mediated *O*-GlcNAc-modified substrates. For the first time, we used the methodical approach that reduced the impact of ncOGT for the benefit of an increased probability of identifying the mOGT’s protein partners and substrates. The results allowed us to specify a group of over 600 proteins as interactors and substrates for mOGT. The identified interacting proteins only partially overlap with the list of *O*-GlcNAcylated proteins from the same cell line. This may result from the way that the samples were prepared for each of the approaches, and especially that the information collected for each of them is different. Thus, protein–protein interactions are generally labile and transient, so many mOGT partners may not have been identified. A cross-linking strategy could help remedy this problem. The anti-*O*-GlcNAc antibody used, RL2, does not specifically recognize all *O*-GlcNAcylated proteins; thus many of the mitochondrial proteins modified by *O*-GlcNAc may not have been enriched and thus identified. Lastly, *O*-GlcNAcylation is also labile and unstable, since it can be quickly removed by OGA. On the other hand, a major positive point is that identified proteins were partially common between the different cell lines, reinforcing the specificity of our strategy. It should be noted that we did not expect a more significant coverage of candidates identified between the cell line because of the disparity of the proteomes. However, we indicated a group of 107 proteins that were present at least in two of three cell lines and were both identified as mOGT interactors and *O*-GlcNAc-bearing proteins. Many of these proteins have been previously proposed as *O*-GlcNAc-modified ones [16,27]. Our analysis revealed that mOGT interacts with and modifies the proteins participating in a variety of mitochondrial processes, such as transport, respiration, amino acid metabolism, protein translation, fatty acid metabolism, apoptosis, and mtDNA processes (Figure 5C and Figure 6). Interestingly, despite the confirmed purity of the mitochondria-enriched fractions, we found a set of 73 candidates identified as nuclear proteins (Table S5). We cannot exclude the possibility that the presence of nuclear proteins among mOGT interactors is a result of mOGT leakage to the nucleus due to its over-expression. However, most of these proteins were identified by two different approaches: the HaloLink^TM^ Resin and the RL2 antibodies. Thus, they were both identified as interactors and *O*-GlcNAcylated proteins. Most of the characterized proteins are associated with chromatin condensation and DNA repair. A previous study by Lazarus and coworkers [20] reported certain nuclear-localized proteins as mOGT substrates. Interestingly, the characterized stretch of three amino-acid (DFP; residues 451–453) as the nuclear localization signal of ncOGT occurs in a region common to that of mOGT [37]. However, to date, the nuclear localization of mOGT has never been observed. Therefore, by analogy to mitochondrial proteins *O*-GlcNAcylated by ncOGT, we suppose that some nuclear proteins may be modified by mOGT before being directed to the nucleus, however, this hypothesis must be validated in the future. 

## 5. Conclusions

Protein *O*-GlcNAcylation is rapidly emerging as a key regulator of critical biological processes. In this study, we focused on the role of poorly explored mitochondrial OGT isoform. We found that the expression of mOGT in breast cancer cells is glucose-dependent. Increased mOGT expression affects mitochondria function and cellular bioenergetics in breast cancer cells. It seems that mOGT dysregulation may be responsible for changes in cellular energy metabolism via interaction with and *O*-GlcNAcylation of a large number of mitochondrial proteins that participate in many processes (Figure 6). In addition, our results suggest that mOGT modifies a specific set of nuclear proteins that deserves to be investigated in the near future.

## Data Availability

The data presented in this study are available in this article (and Supplementary materials).

## Supplementary Materials

The following are available online at https://www.mdpi.com/article/10.3390/cancers13122956/s1. Table S1: mOGT Interactors, Table S2: *O*-GlcNAcylated candidates, Table S3: Both method per cell line, Table S4: Gold list, Table S5: Nuclear candidates, Table S6: All mitochondrial Candidates, File S1. The original western blot figures, File S2: Supplementary methods, Figure S1: Densytometric analysis of O-GlcNac levels in mitochondria-enriched fraction from Western blots presented on Figure 2B, Figure S2: mOGT up-regulation alter cellular respiration and glycolytic activity, Figure S3: Intracellular and mitochondrial calcium levels in breast cancer cells with up-regulated mOGT, Figure S4: mOGT treated cells lose the viability and change their cell cycle.
