# Supplementary material for: Mitochondrial O-GlcNAc Transferase Interacts with and Modifies Many Proteins and Its Up-Regulation Affects Mitochondrial Function and Cellular Energy Homeostasis
Source: Cancers (Basel). 2021 Jun 12;13(12):2956. doi: 10.3390/cancers13122956 (PMC8231590; doi:10.3390/cancers13122956)
Supplement: Supplementary file 1 [file cancers-13-02956-s001.zip › File S2. Supplementary Methods.pdf]

cancers

MDPI

Mitochondrial *O*-GlcNAc Transferase Interacts with and Modifies Many Proteins and Its Up-Regulation Affects Mitochondrial Function and Cellular Energy Homeostasis

Paweł Jóźwiak, Piotr Ciesielski, Piotr K. Zakrzewski, Karolina Kozal, Joanna Oracz, GrażynaBudryn, Dorota Żyżelewicz, StéphanieFlament, Anne-Sophie Vercoutter-Edouart, Fabrice Bray, Tony Lefebvre and Anna Krześlak

1. RT-PCR

Table: Detailed information concerning the used primers.

Gene	Sequence	Tm° range	Product length
mOGT	5′-tggctggtcagagaaggaataa-3′<br>3′-gacgttggatcggttacgag-5′	63,8° – 67,7°	511
ncOGT	5′-gcaacctagccaatgctctc-3′<br>3′-cagaagggtctcaaacgacg-5′	63,5° – 70,1°	196
GAPDH	5′-ccctggcgtcgtgattagtg-3′<br>3′-cctgacttgcagaacgagct-5′	65,1° – 70,1°	176
HPRT1	5′-cctgcaccaccaactgctta-3′<br>3′-aggtcttgtagtagggacgg-5′	60,4° – 69,9°	139

2. Mitochondrial Oxygen Consumption Assay

Mitochondrial respiration was measured in live cells using Extracellular O_2 Consumption Assay (Abcam, Cambrige, UK) as detailed in the manufacturer's manual. The assay is based on the ability of oxygen to quench the excited state of Extracellular O_2 Consumption reagent in cell growing media. As the mitochondria consume oxygen during cellular respiration, oxygen is depleted in the media, which is seen as an increase in phosphorescence signal. To limit back diffusion of ambient oxygen into media each well of plate was covered by a high-sensitivity mineral oil. Cells were seeded on the plate wells at a density of 6×10^4 cells/well for MCF-7 or 4×10^4 cells/well for MDA-MB-231 and Hs578t in 200 μL culture media. The next day, cells were transfected for 48 h with plasmid DNA as described in the cell culture and treatment section. After this time, the culture medium from all assay wells was removed and replaced with 150 μL of fresh culture media and then, 10 μL of reconstituted Extracellular O_2 Consumption Reagent was added. Each well was promptly sealed by dropping 100 μL of pre-warmed High Sensitivity mineral oil. Extracellular O_2 Consumption signal was measured at 1.5 min. intervals for 4 hours at Ex/Em = 380/650 nm using pre-warmed (37°C) Bio-Tek Synergy HT Microplate Reader (Bio-Tek Instruments, Winooski, VT, USA).

3. Glucose Uptake Measurement

Glucose uptake was evaluated by flow cytometry using the fluorescent glucose analog 2-(N-[7-nitrobenz-2-oxa-1,3-diazol-4-yl]amino)-2-deoxyglucose (2-NBDG) (Molecular Probes, Eugene, OR, USA) [1]. Cells grown for 24 or 48 h in medium containing 2, 5, or 25 mM glucose were washed with PBS and a medium containing 0.25 mM glucose and 300 μM 2-NBDG was added. After 1 h incubation, cells were washed two times with PBS, trypsinized, centrifuged, and resuspended in PBS. Optical filters of the flow cytometer were set up such that 2-NBDG was measured using the green fluorescence (520 nm) channel FL1. The results were collected as a median fluorescent signal from a population of 10^4 cells.

4. Lactic Acid Assay

The Lactate Assay Kit (Sigma-Aldrich, St. Louis, MO, USA; cat. nr. MAK064) was used to measure L(+)-Lactate concentration in breast cancer cells or post-culture media. The measurements were performed according to the manual. Cell growing in 12-well plates under various glucose concentrations were transfected with plasmid DNA for 48 hours. In order to determine lactate acid excreted from the cells, two hours before cells collection, the complete medium containing lactate dehydrogenase (LDH) was removed and monolayers were washed twice with DPBS, then 0.4 mL of Phenol Red free medium without FBS was added into each well. Cell pellets or 50 µL of post-culture media were mixed by pipetting up and down in 4 volumes of the Lactate Assay Buffer. To remove insoluble material, the lysed samples were centrifuged at 13,000 ×*g* for 10 minutes followed by transfer supernatants to the clean vials. Simultaneously, the standards containing from 0 to 100 pmol of lactic acid were prepared. Then, 50 µL of each sample was placed into a 96-well plate (µClear®, black, flat bottom; Greiner Bio-One GmbH, Frickenhausen, Germany). The reaction was started by adding 50 µL of the Master Reaction Mix to each of the wells. Plate was mixed using a horizontal shaker and incubated for 30 min in the dark, at room temperature. Fluorescence intensity (λ_{ex}=535/λ_{em}=587 nm) was measured using a Bio-Tek Synergy HT Microplate Reader (Bio-Tek Instruments, Winooski, VT, USA). All reactions were performed in triplicate. Lactic acid concentration in samples was calculated from standard curve.

5. Calcium Levels Measurement

Cells were seeded on the plate wells at a density of 6×10^4 cells/well for MCF-7 or 4×10^4 cells/well for MDA-MB-231 and Hs578t in 200 µL culture media. The next day, cell growing in 96-well plates under various glucose concentrations were transfected with plasmid DNA for 48 hours. as described in the cell culture and treatment section. Ca^{2+} release into the cytosol was determined by measuring the ratio between Ca2-bound Indo signal (at 405 nm) and Ca2-unbound Indo signal (at 480 nm) using cell permeant Indo dye (Molecular Probes™, Eugene, OR, USA). Cells were labeled for 20 min at 37°C with pre-warmed calcium-free medium containing Indo-1 at a final concentration 1 µM. Then, cells were washed twice with PBS and incubated for the further 30 minutes to allow complete de-estrification of intra-cellular AM esters. The fluorescence shift ratio was determined using a Bio-Tek Synergy HT Microplate Reader (Bio-Tek Instruments, Winooski, VT, USA). Mitochondrial Ca^{2+} calcium level was measured by cell permeant Rhod-2 indicator (Molecular Probes™, Eugene, OR, USA). Cells were stained for 30 min at 37°C with pre-warmed calcium-free medium containing Rhod-2 at a final concentration 2,5 µM. Next, cells were washed twice with PBS and incubated for 24 h at 37°C with fresh culture medium to eliminate cytosolic staining produced by Rhod-2. After this time, the fluorescence intensity at 580 nm corresponding to mitochondrial calcium was measured by a Bio-Tek Synergy HT Microplate Reader (Bio-Tek Instruments, Winooski, VT, USA).

6. Cell Viability and Proliferation Assessment

Cell viability was assessed using the MTT assay. Cells were plated onto 96-well plates at a density of 10.000 cells for MDA-MB-231 and Hs578t, and 15.000 cells for MCF-7, then cultured in standard conditions. After 24 h, the medium was replaced with fresh medium and the cells were treated with different glucose concentrations and transfected with plasmid DNA for next 24 or 48 h. Twenty µL of 0.05% (*m*/*v*) MTT [3-(4,5-dimethylthiazol-2-yl)-2,5 diphenyltetrazolium bromide] (Sigma-Aldrich, St. Louis, MO, USA) in medium was added to each well and the cells were incubated for 2 h in 37°C. The medium was then removed and 50 µl of DMSO was added. The absorbance of the formazan product was measured at 590 nm on a Bio-Tek Synergy HT Microplate Reader (Bio-Tek Instruments, Winooski, VT, USA) with a blank (medium alone) as the background control.

Cell proliferation was analyzed based on BrdU incorporation according to published protocol [2]. Cells were pulse-labeled with 50 μM BrdU (Sigma-Aldrich, St. Louis, MO, USA) for 2 hours. Cells were then washed twice with PBS, trypsinized, and fixed with 70% (*v*/*v*) ethanol at 4ºC for 2 hours. After fixation, the cells were denatured in 2 M HCl-0,5% (*v*/*v*) Triton X-100 for 30 min and washed once in 0.1 M sodium tetraborate for 2 min. Then, cells were washed once in PBS-1% (*m*/*v*) BSA and resuspended in 1μg/mL anti-BrdU antibody (Abcam®, Cambridge, UK) in PBS-1% (*m*/*v*) BSA for 1 h, followed by three washes with 0,5% (*v*/*v*) Tween 20- 1% (*m*/*v*) BSA in PBS. The cells were incubated with 0.5 μg/mL FITC-conjugated secondary antibody in PBS-1% (*m*/*v*) BSA for 30 min, then counterstained for 45 min with 20 μg/mL propidium iodide in PBS containing 10 μg/mL RNAse A. The stained cells were detected using an LSR II flow cytometer (BD Biosciences), and then analyzed with FlowJo v9.9.4 software (TreeStar, Ashland, OR, USA).

References

1. Yamada, K., Saito, M., Matsuoka, H., Inagaki, N. A real-time method of imaging glucose uptake in single, living mammalian cells. *Nat. Protoc.***2007**, *2*, 753–762, doi: 10.1038/nprot.2007.76.
2. Cecchini, M.J.,Amiri, M.,Dick F.A.Analysis of Cell Cycle Position in Mammalian Cells. *J. Vis.Exp.***2012,***59*, e3491, doi: 10.3791/3491.
