# Supplementary material for: Mitochondrial O-GlcNAc Transferase Interacts with and Modifies Many Proteins and Its Up-Regulation Affects Mitochondrial Function and Cellular Energy Homeostasis
Source: Cancers (Basel). 2021 Jun 12;13(12):2956. doi: 10.3390/cancers13122956 (PMC8231590; doi:10.3390/cancers13122956)
Supplement: Supplementary file 1 [file cancers-13-02956-s001.zip › Supplementary Figures S1.-S4..pdf]

cancers

MDPI

Mitochondrial *O*-GlcNAc Transferase Interacts with and Modifies Many Proteins and Its Up-Regulation Affects Mitochondrial Function and Cellular Energy Homeostasis

Paweł Jóźwiak, Piotr Ciesielski, Piotr K. Zakrzewski, Karolina Kozal, Joanna Oracz, GrażynaBudryn, Dorota Żyżelewicz, StéphanieFlament, Anne-Sophie Vercoutter-Edouart, Fabrice Bray, Tony Lefebvre and Anna Krześlak

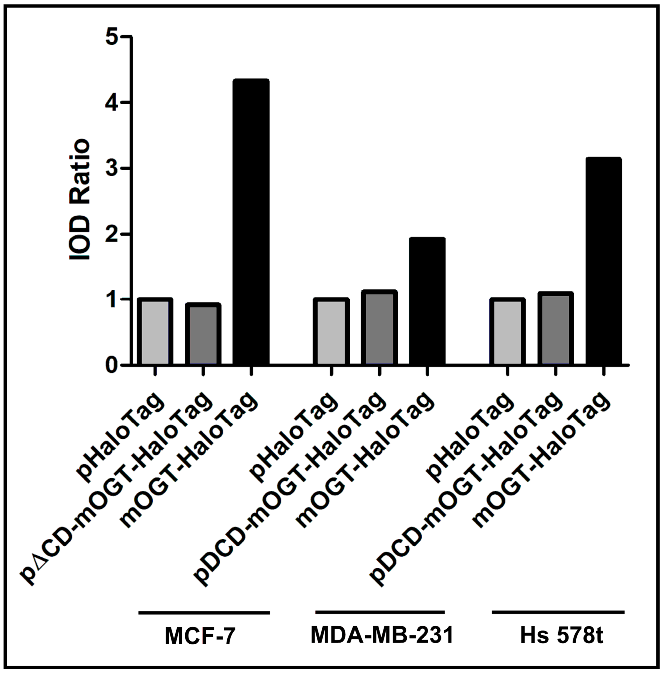

Figure S1. Densytometric analysis of O-GlcNac levels in mitochondria-enriched fraction from Western blots presented on Figure 2B. The analysis was performed using GelPro Analyzes ver. 3.1 software (Media Cybernetics). IOD; integrated optical density.

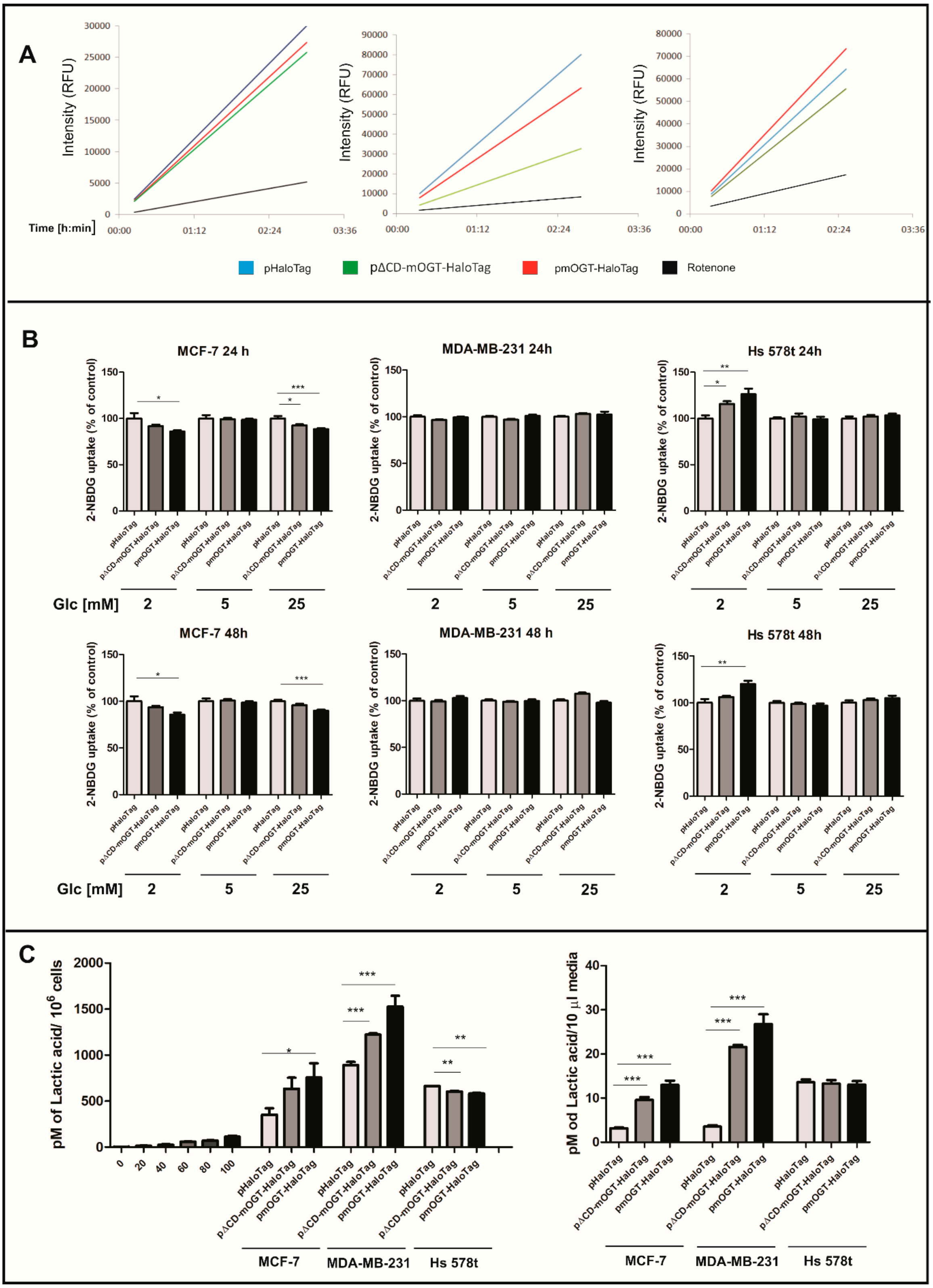

Figure S2. mOGT up-regulation alter cellular respiration and glycolytic activity. (**A**) Mitochondrial respiration in cells transfected for 48 h with HaloTag (blue line) or mOGT-HaloTag (red line). The graph is showing oxygen depletion in the living cells media as an increase in phosphorescence signal intensity of Extracellular O2 Consumption Reagent over time. (**B**) Glucose uptake by cells Table 2. NBDG incorporation into living cells. The results are presented as median fluorescence signal of 2-NBDG detected using flow cytometry. (**C**) Intracellular (a) and post-culture media (b) Fluorometric detection of L(+)-lactic acid concentration in cells treated for 48 h with plasmid DNA.Data represent the average of at least 3 independent experiments performed in tetraplicates. * indicates significance p <0,05; ** p <0,01; *** p < 0,001; Rotenone; an inhibitor of complex I of the mitochondrial respiratory chain.

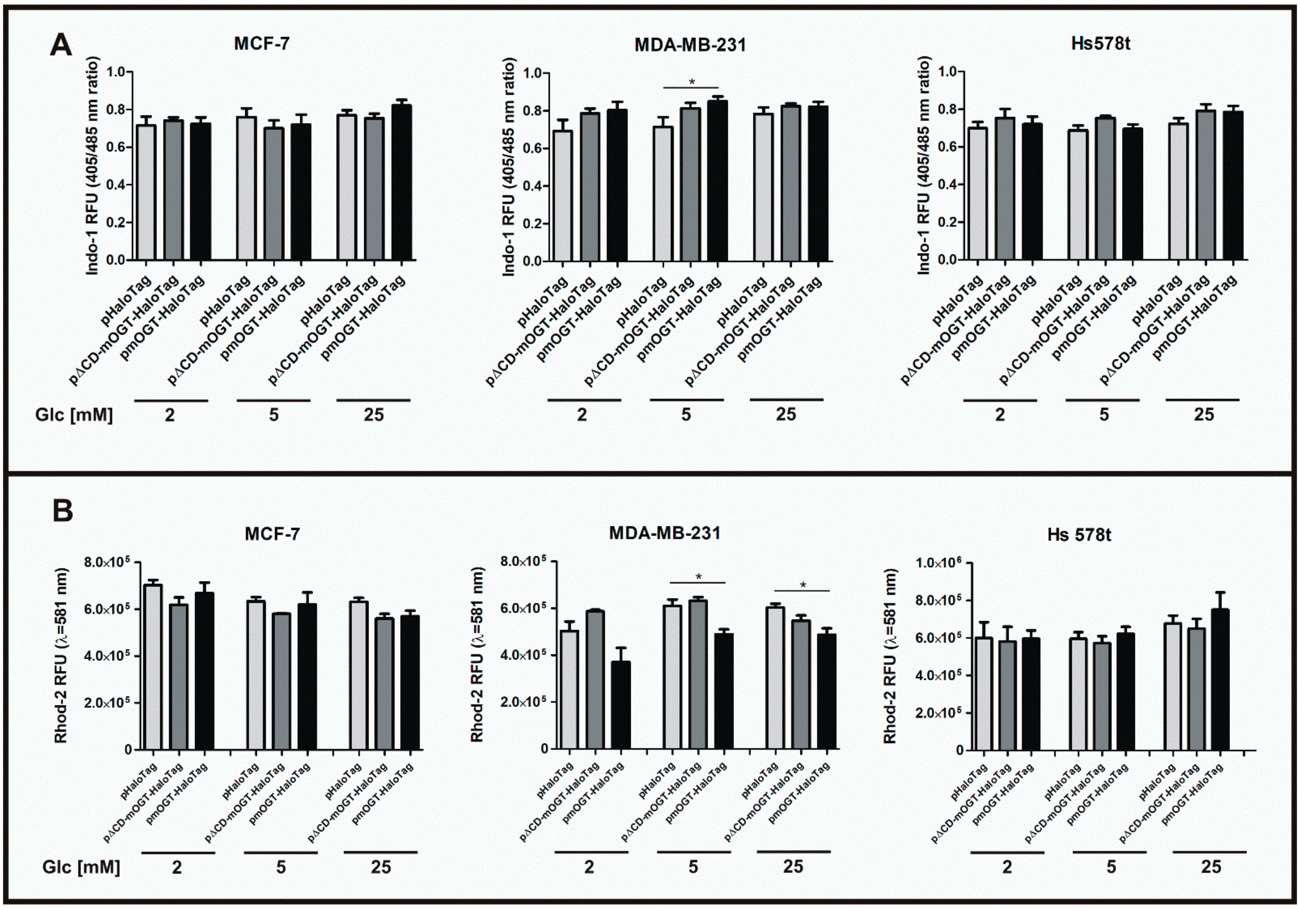

Figure S3. Intracellular and mitochondrial calcium levels in breast cancer cells with up-regulated mOGT. (**A**)Ca2+ release into the cytosol determined by measuring the ratio between Ca2-bound Indo signal (at 405 nm) and Ca2−unbound Indo signal (at 480 nm) (**B**) mitochondrial Ca2+ calcium level measured by Rhod-2 indicator which accumulates in mitochondria. * indicates significance $p < 0,05$. Glc; Glucose.

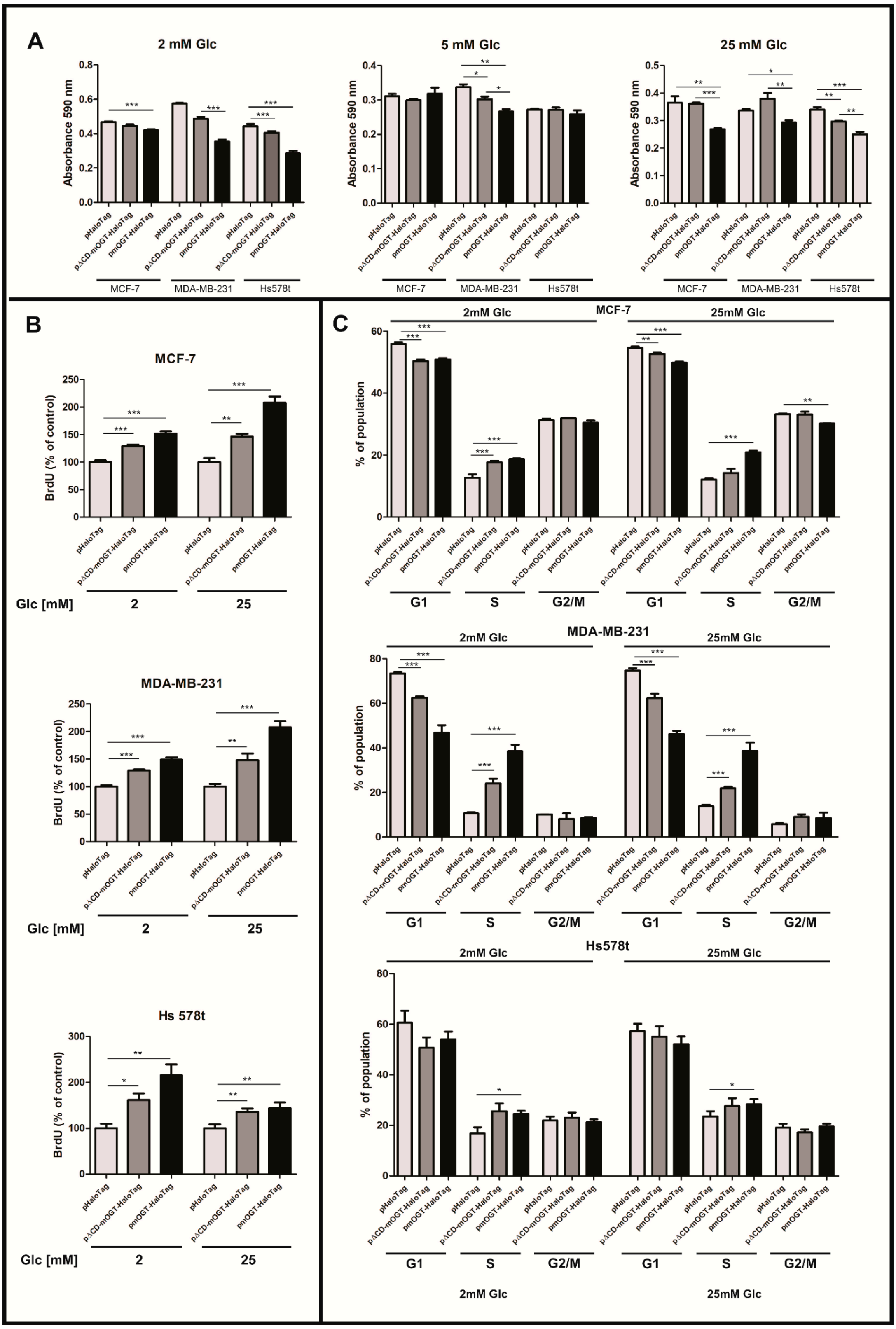

Figure S4. mOGT treated cells lose the viability and change their cell cycle. Cells were growing in different glucose conditions and treated for 48 h with plasmid DNA. (**A**)Viability of cells determined by MTT assay, (**B**) Proliferation of cells measured based on BrdU incorporation into DNA (**C**) Cell population in G1, S and G2/M cell cycle phases assessed by labeling cells with propidium iodide. Data represent the average of at least 3 independent experiments performed in tetraplicates and are expressed as means +/− SD (bar graphs shown in A) or median +/− S.E. (bar graphs shown in B and C). * indicates significance $p < 0,05$; ** $p < 0,01$; *** $p < 0,001$. Glc; Glucose.
