# Supplementary figures and images for: Mitochondrial O-GlcNAc Transferase Interacts with and Modifies Many Proteins and Its Up-Regulation Affects Mitochondrial Function and Cellular Energy Homeostasis
Source: Cancers (Basel). 2021 Jun 12;13(12):2956. doi: 10.3390/cancers13122956 (PMC8231590; doi:10.3390/cancers13122956)

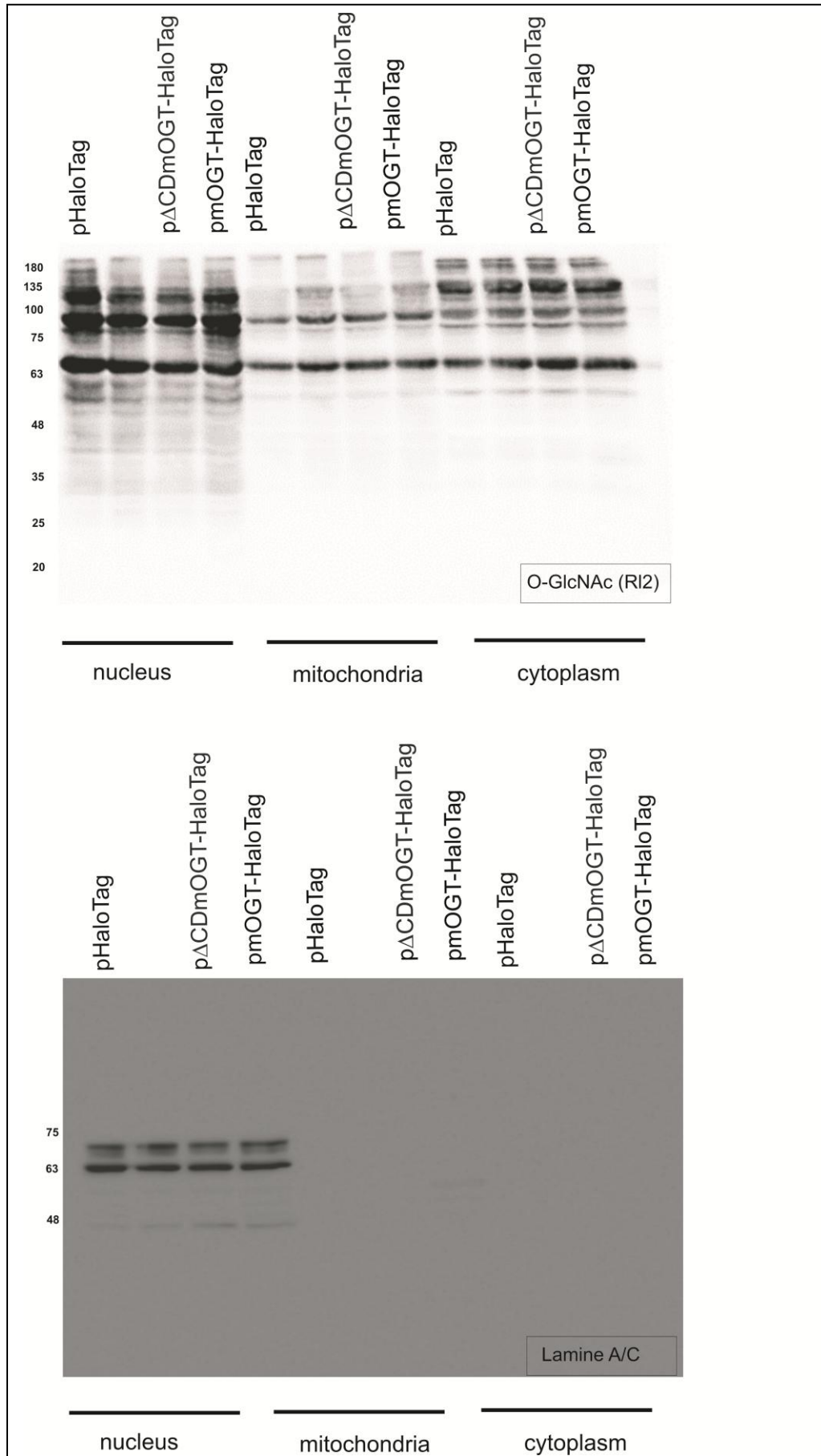

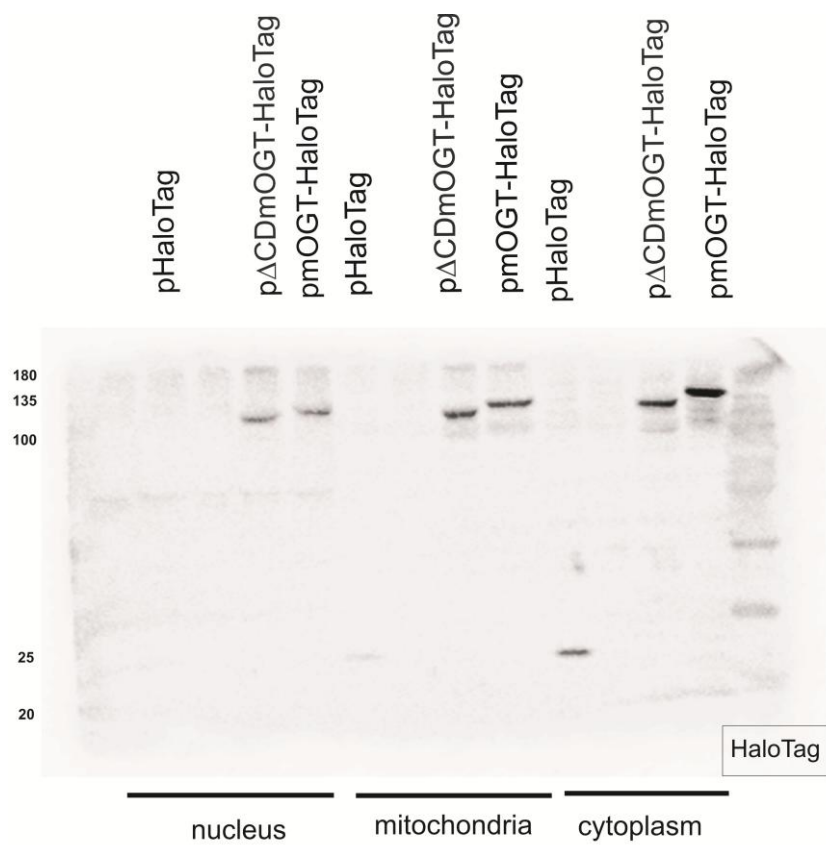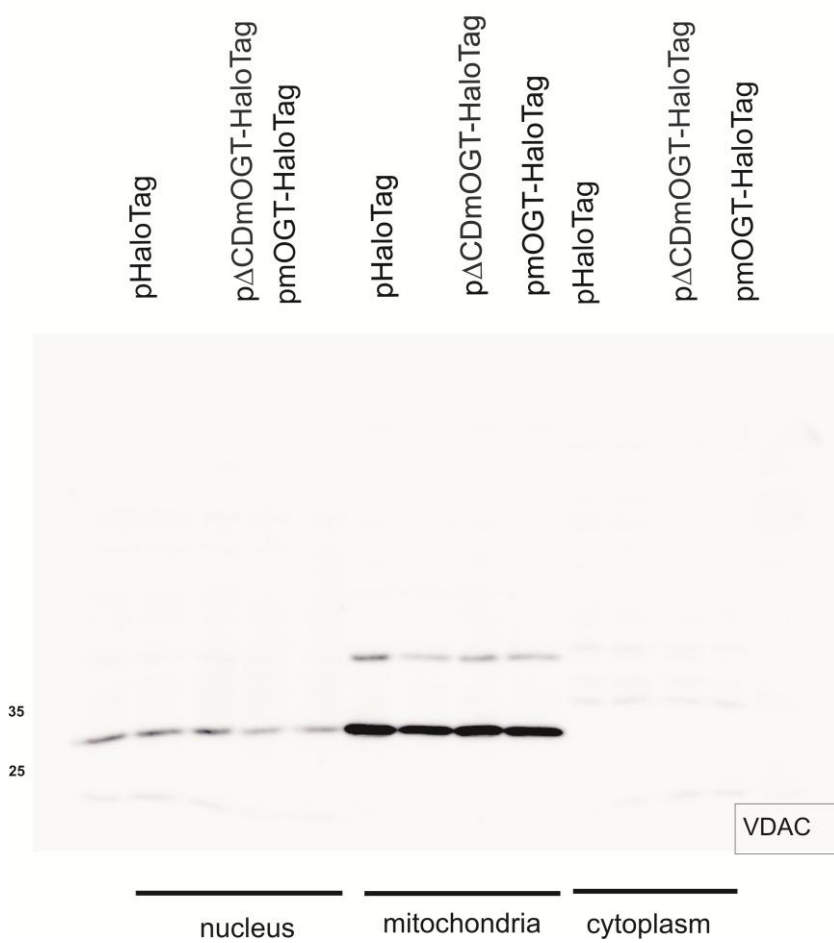

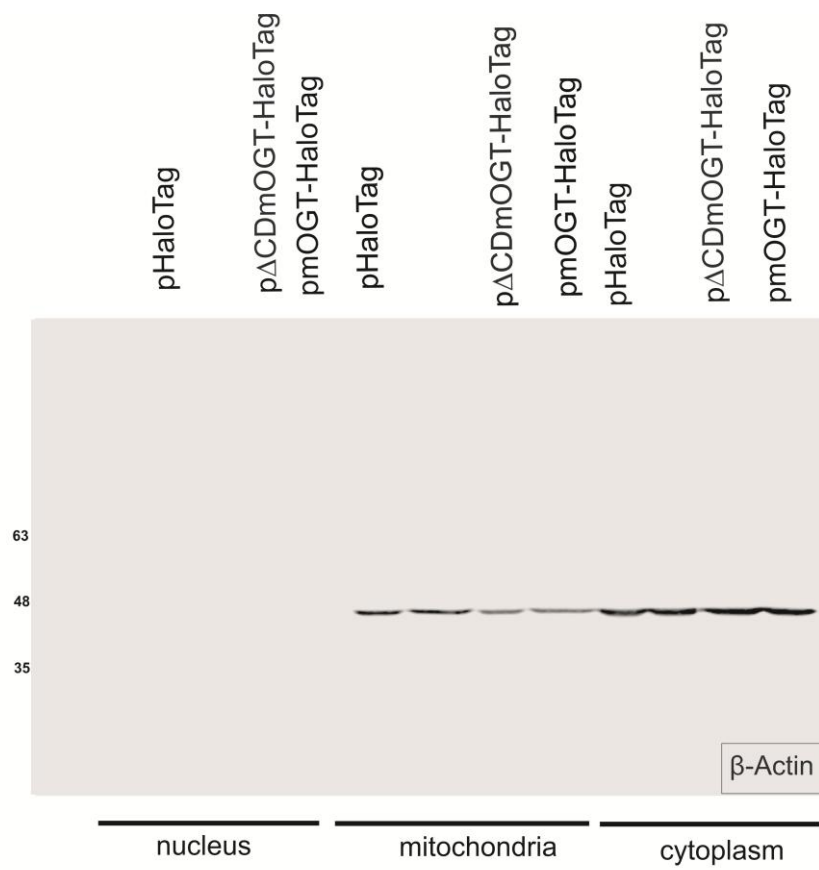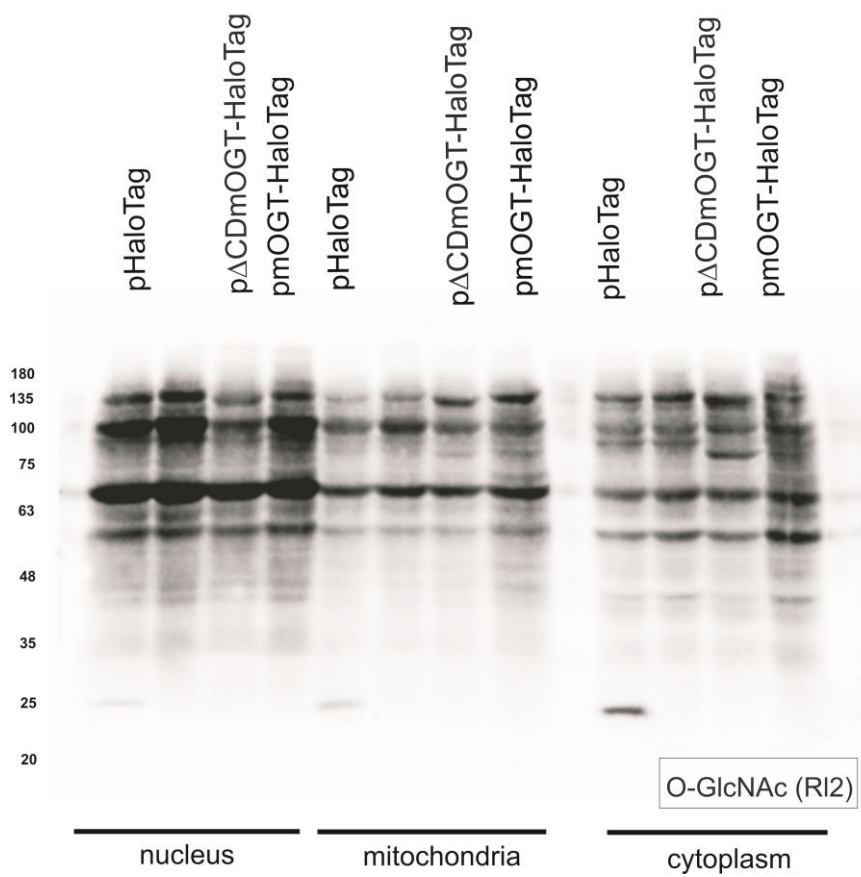

Hs578t

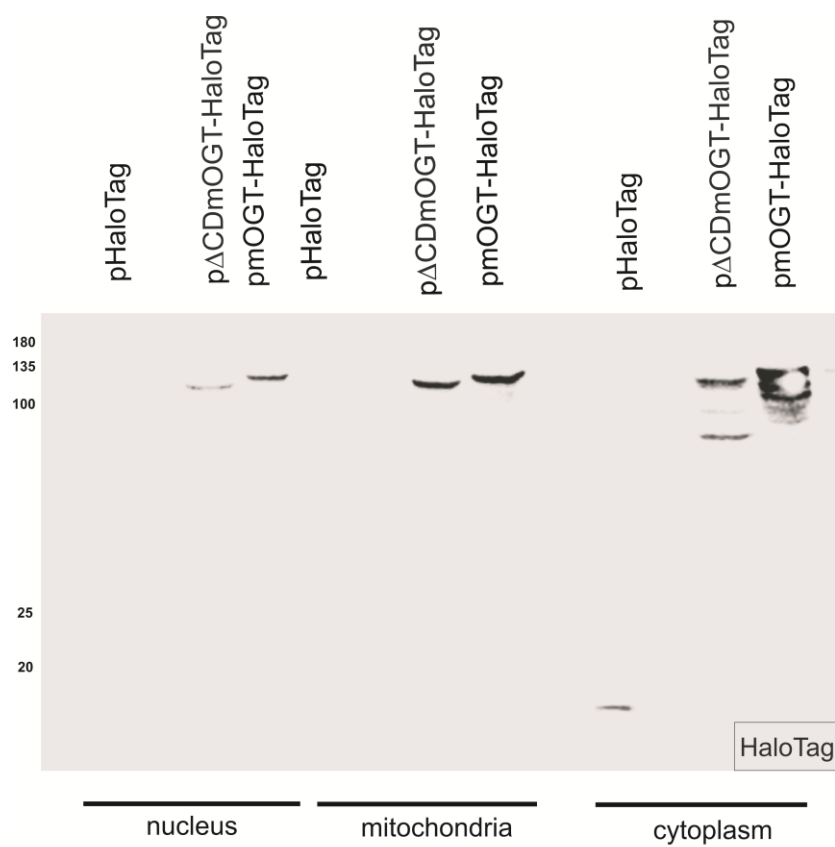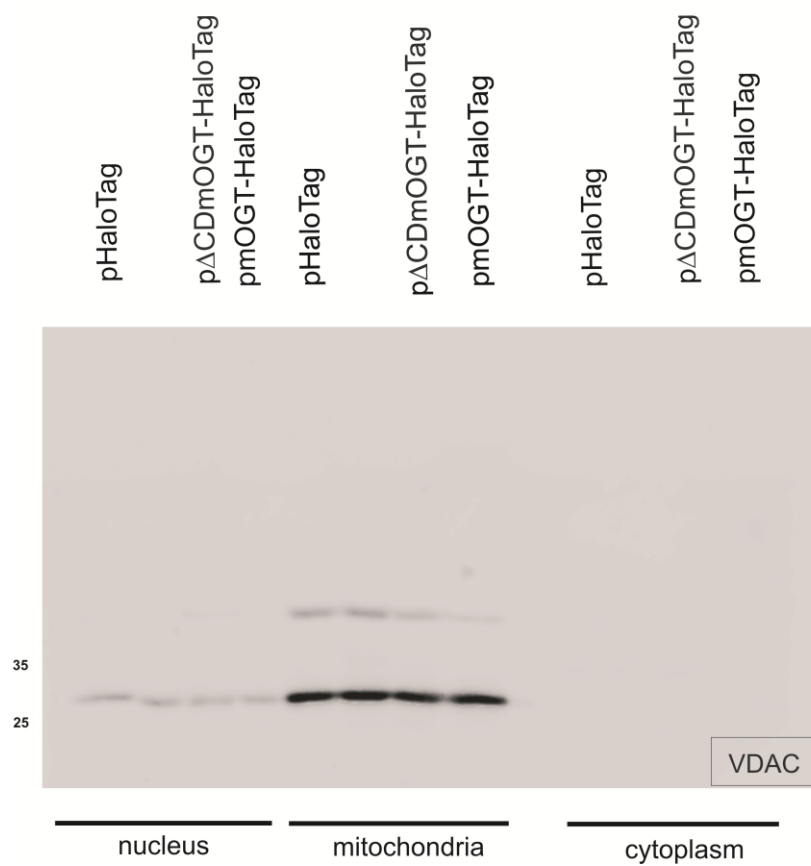

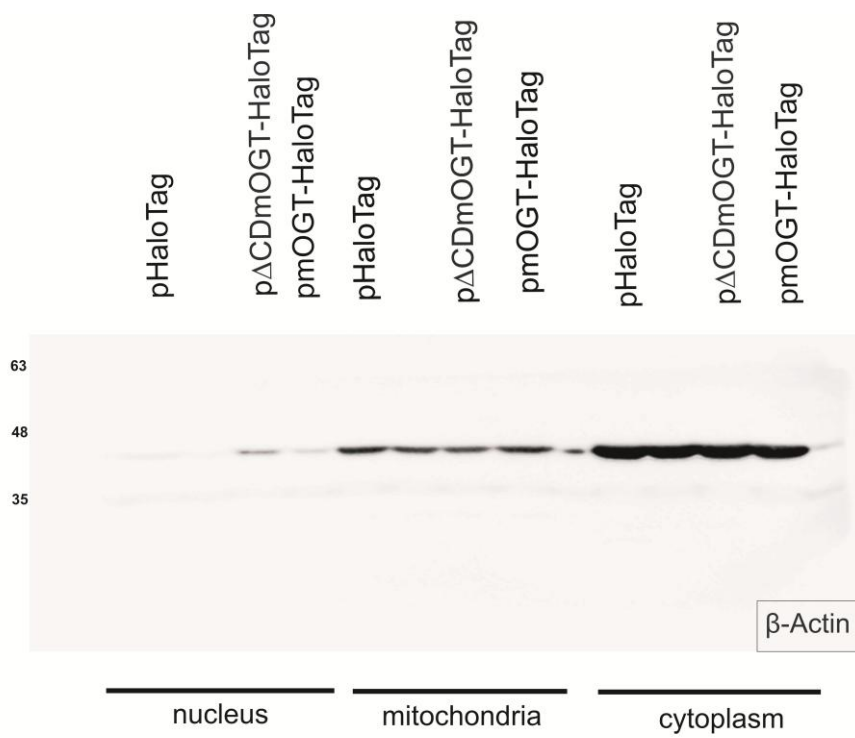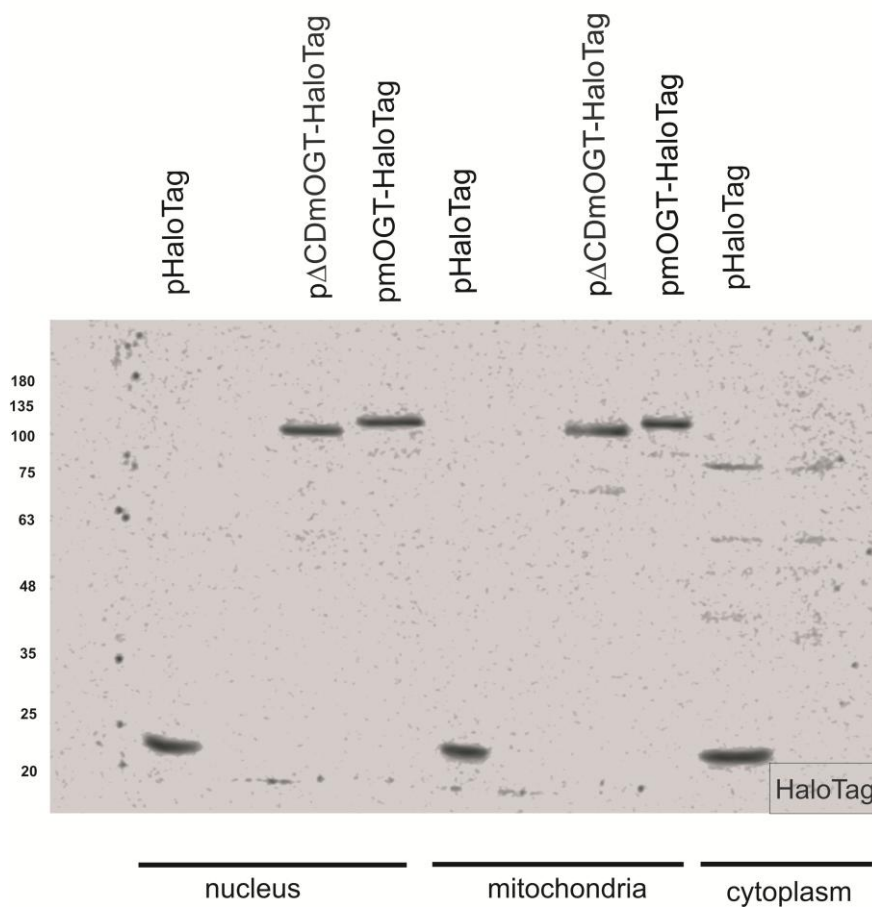

MCF-7

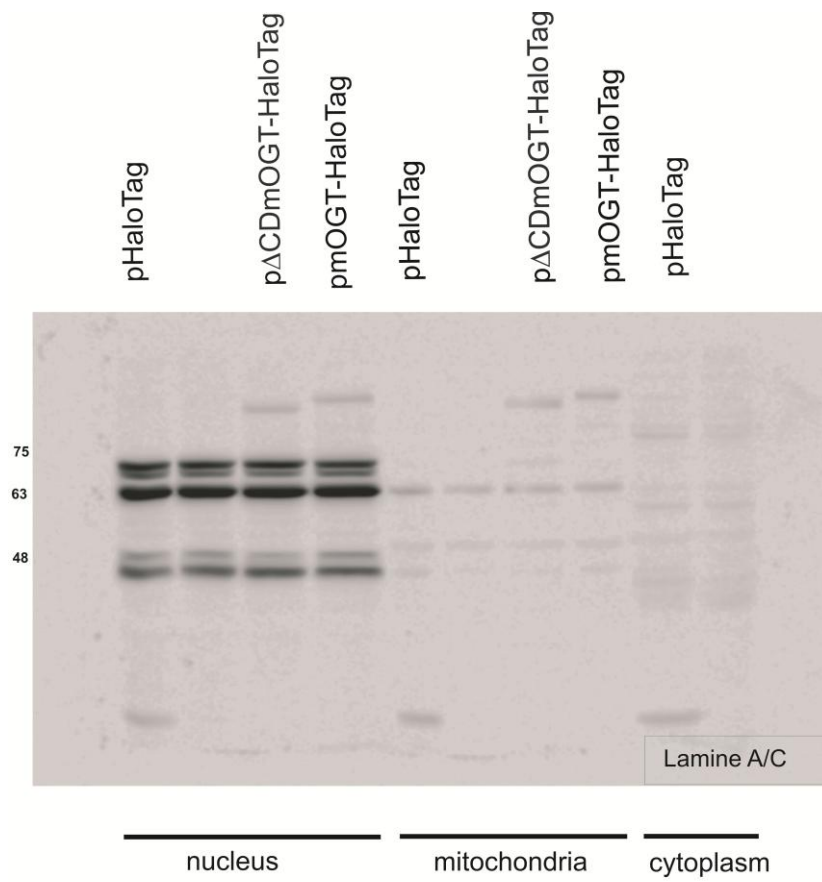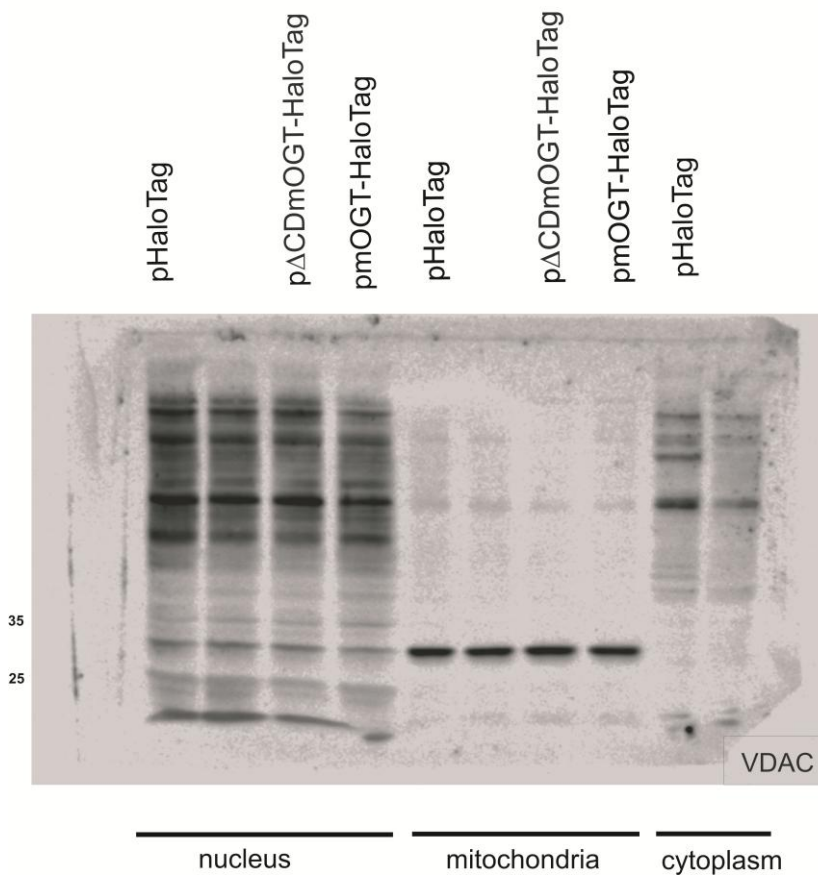

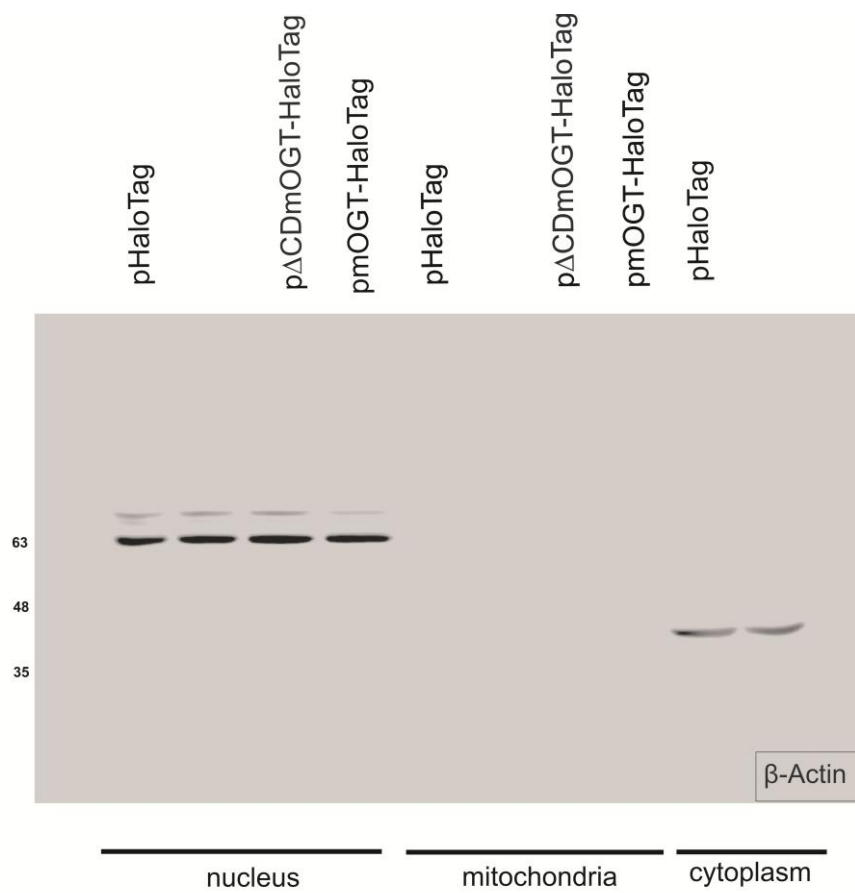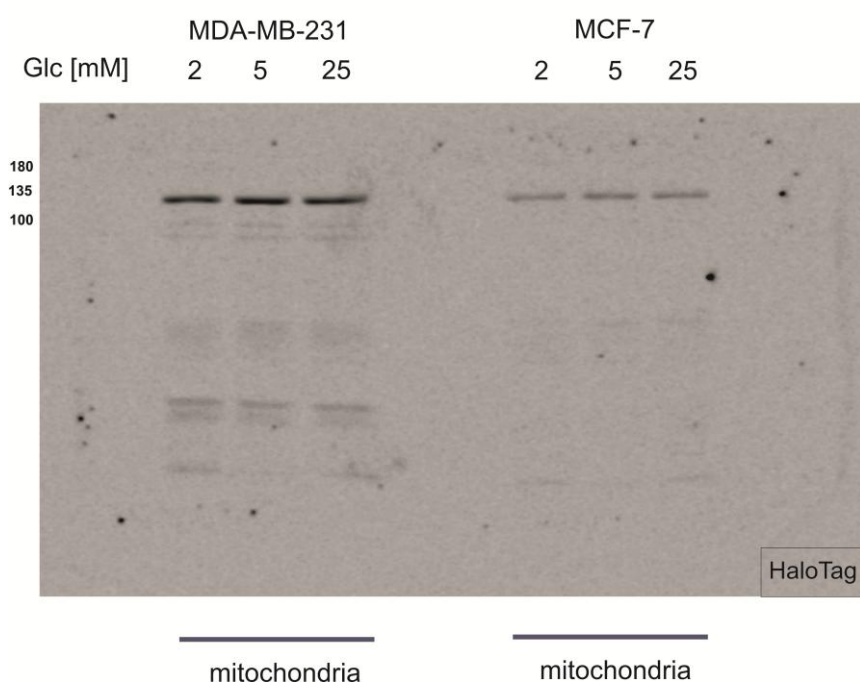

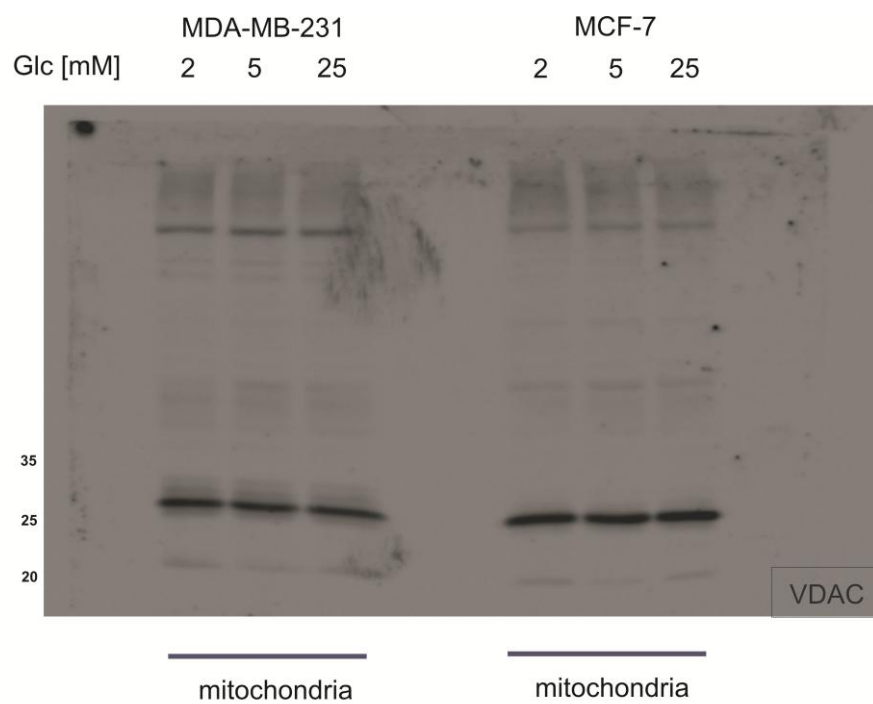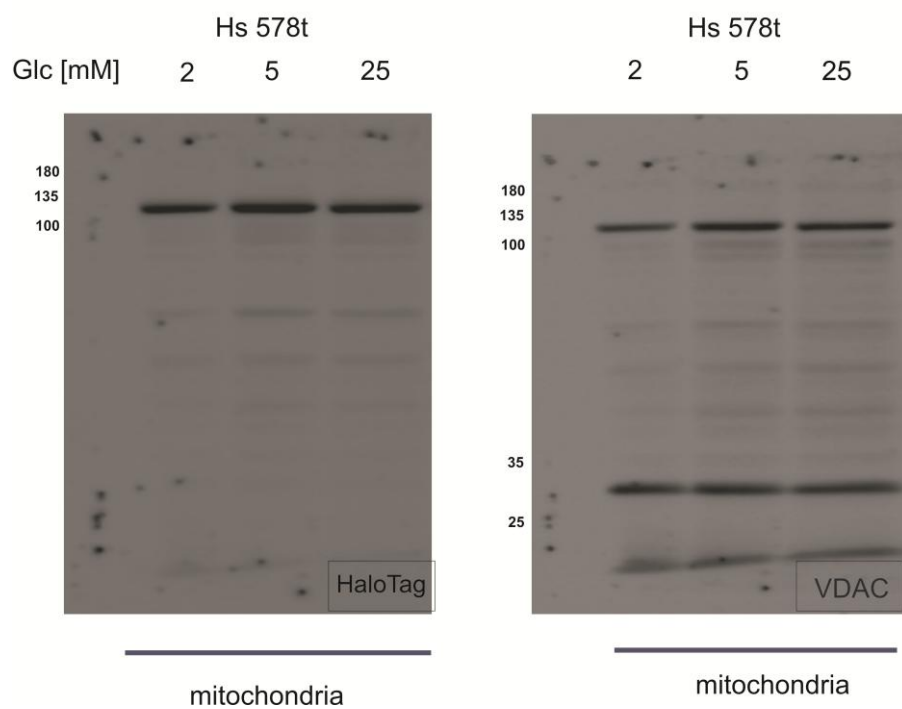

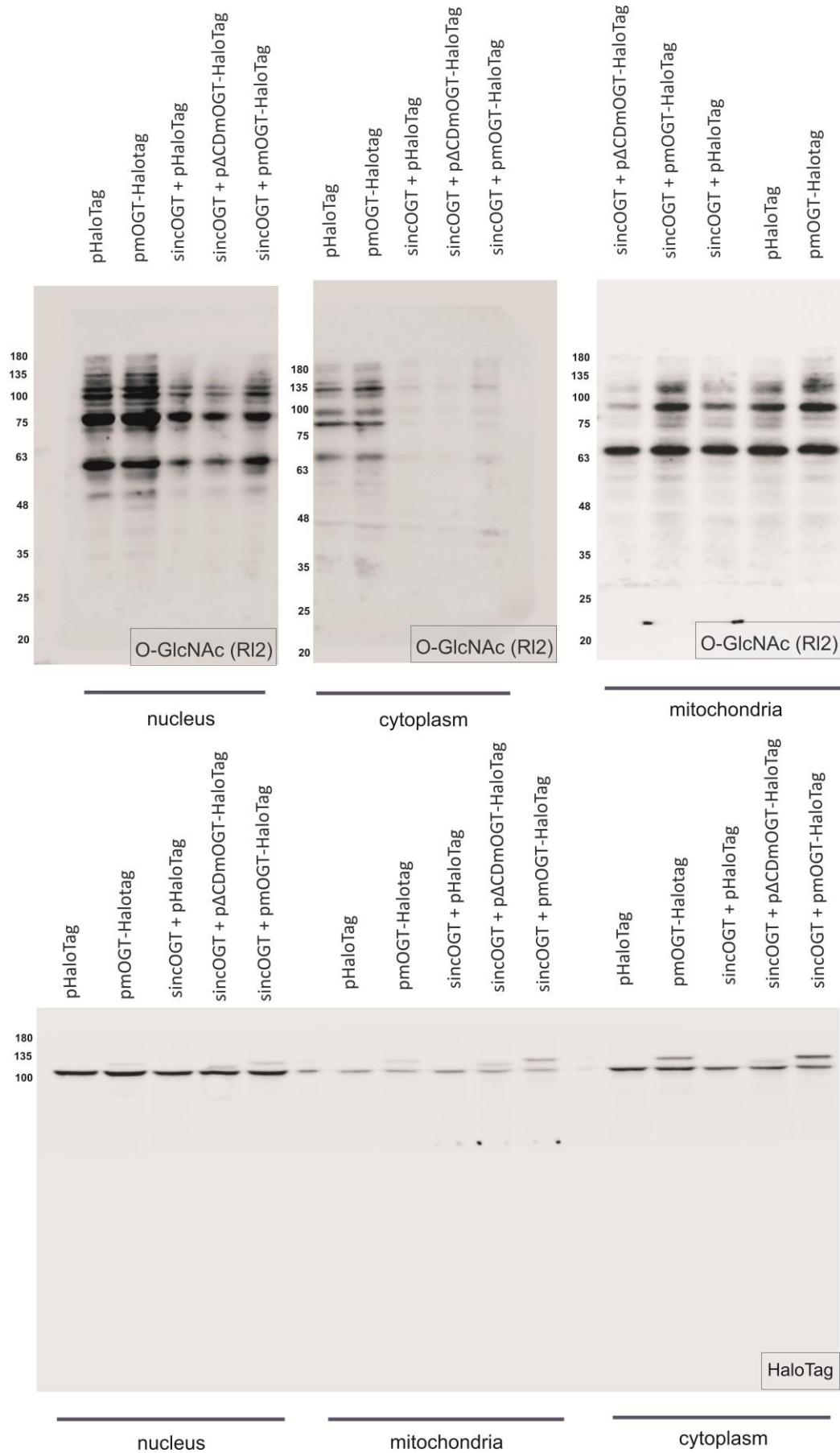

MCF-7

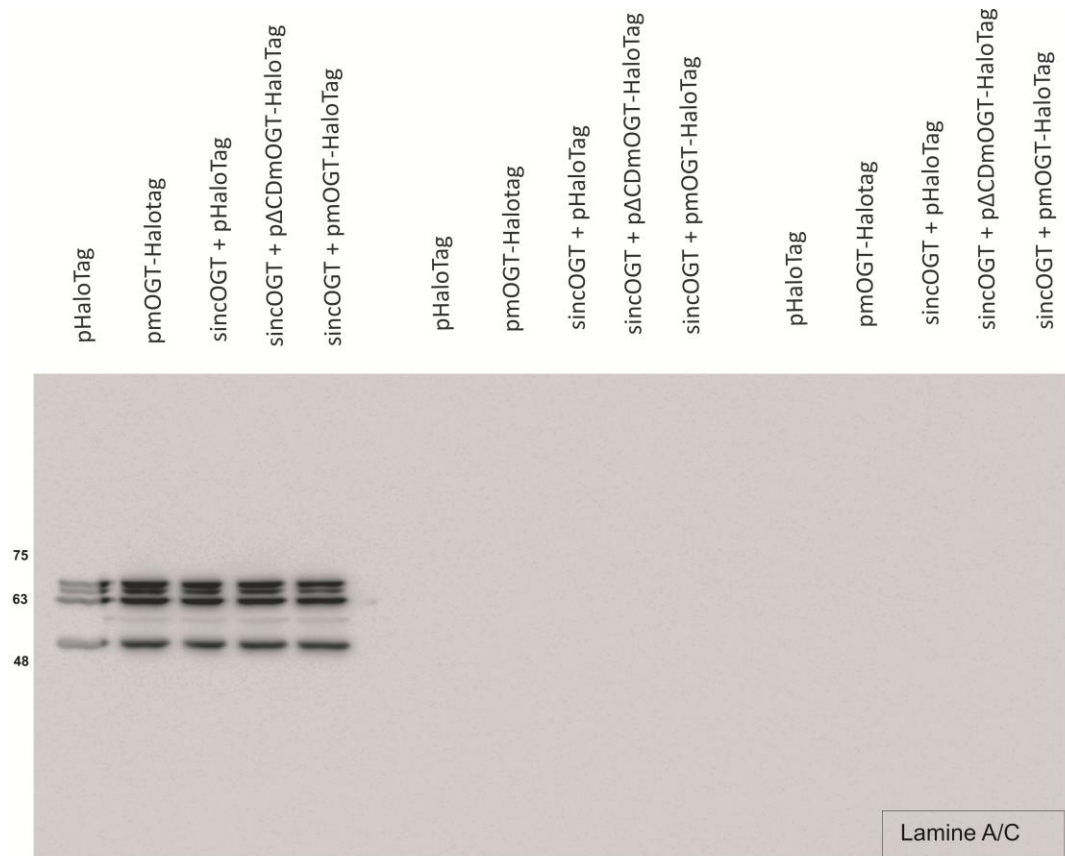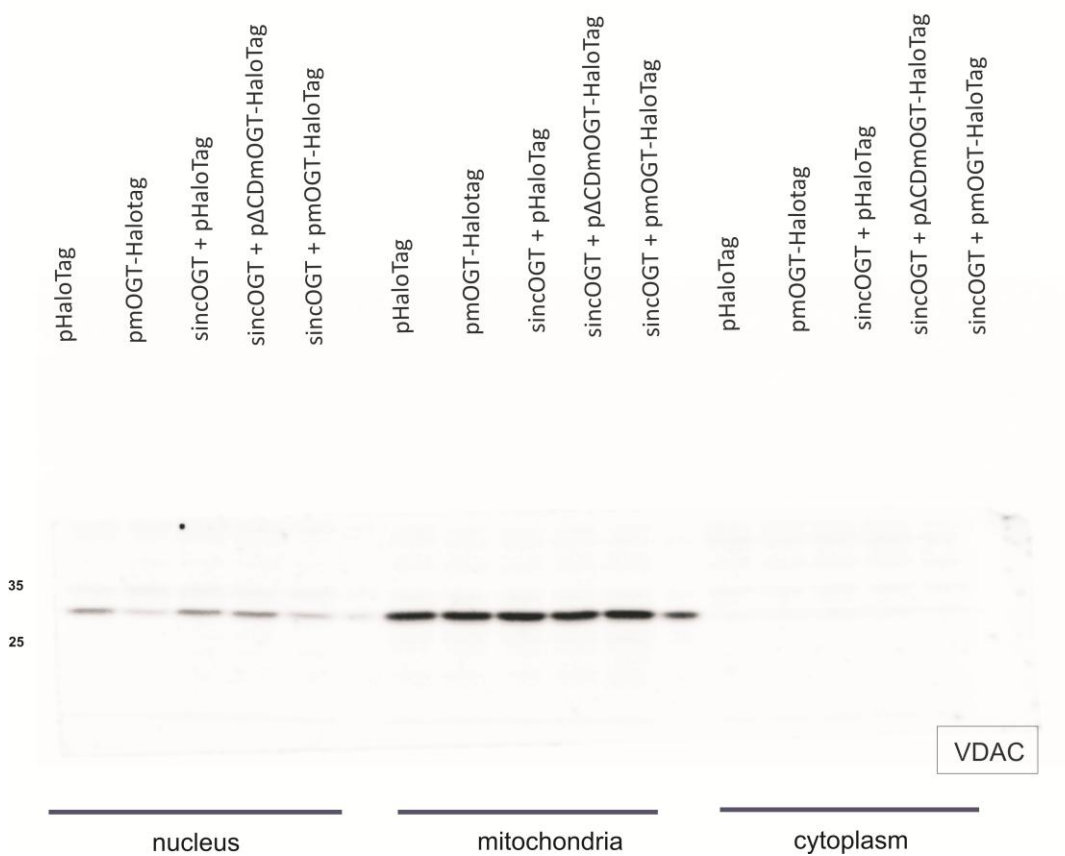

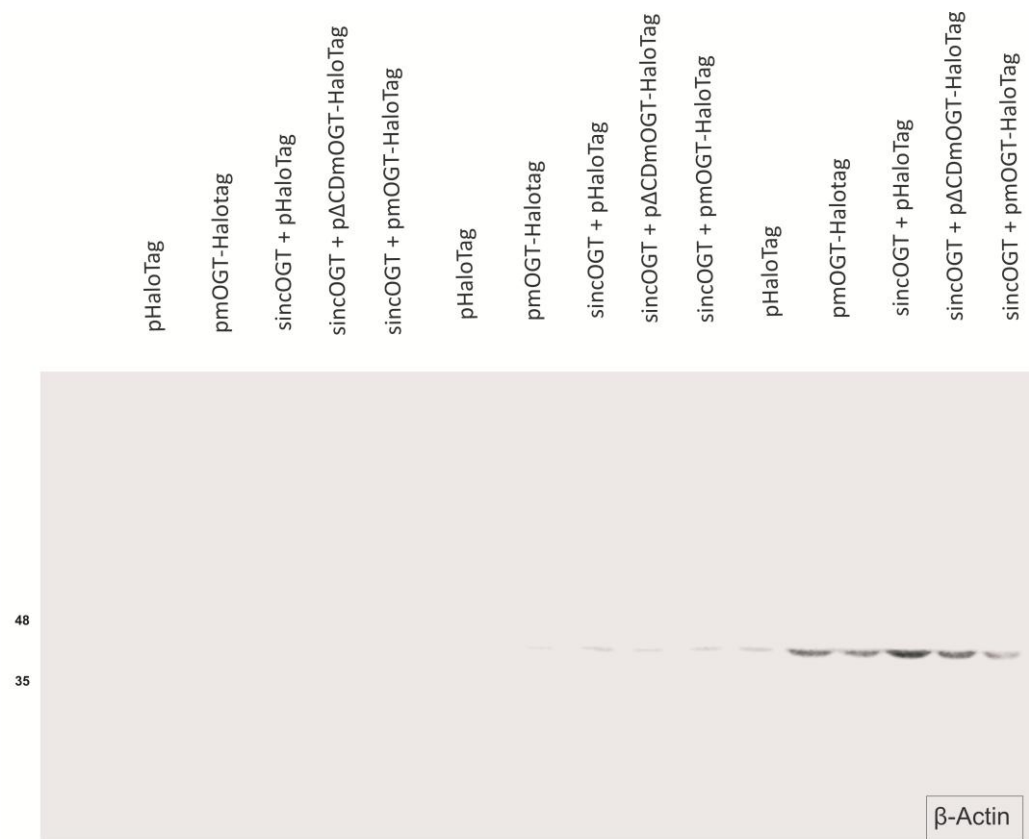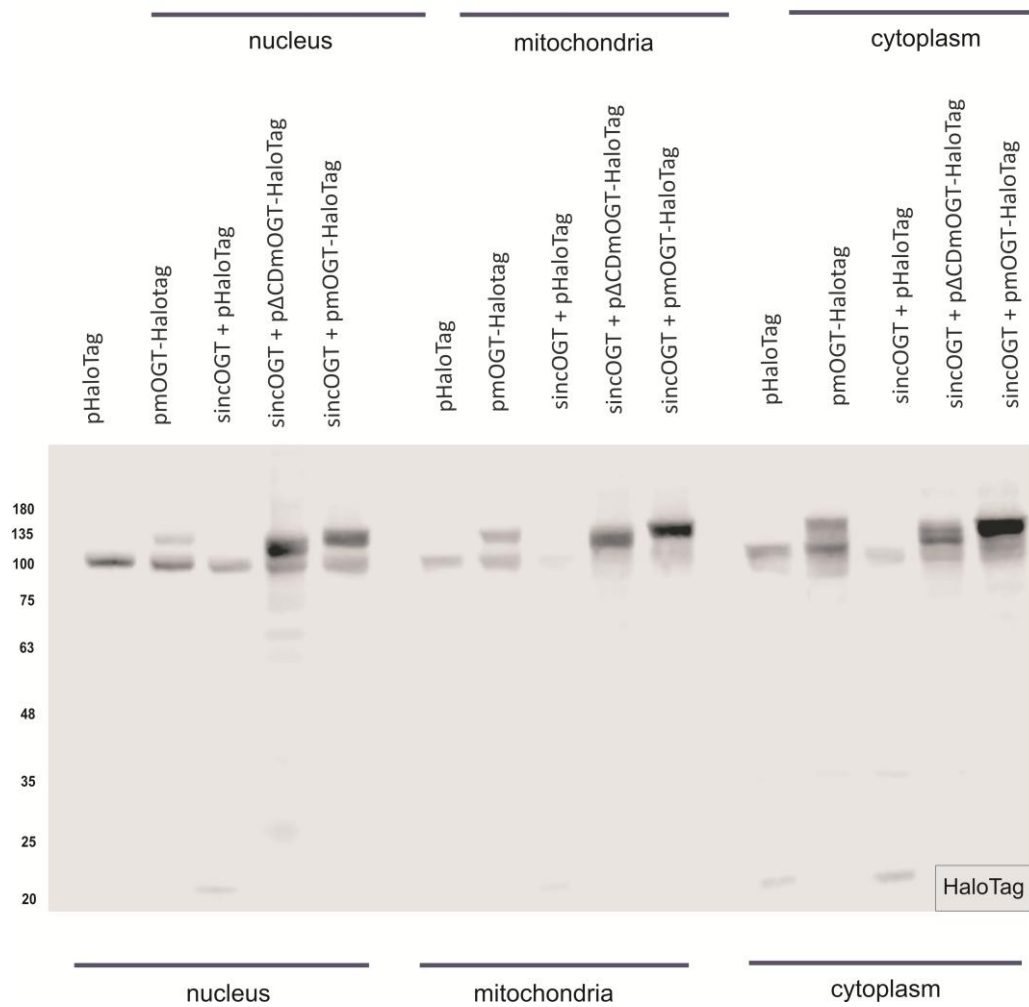

MDA-MB-231

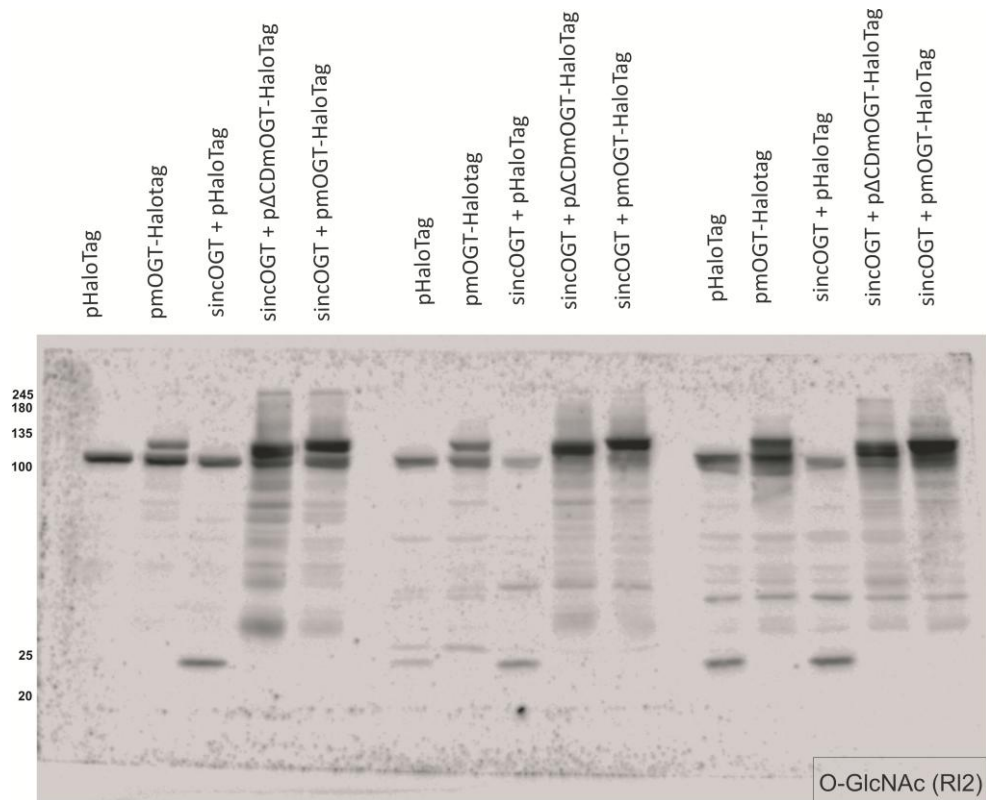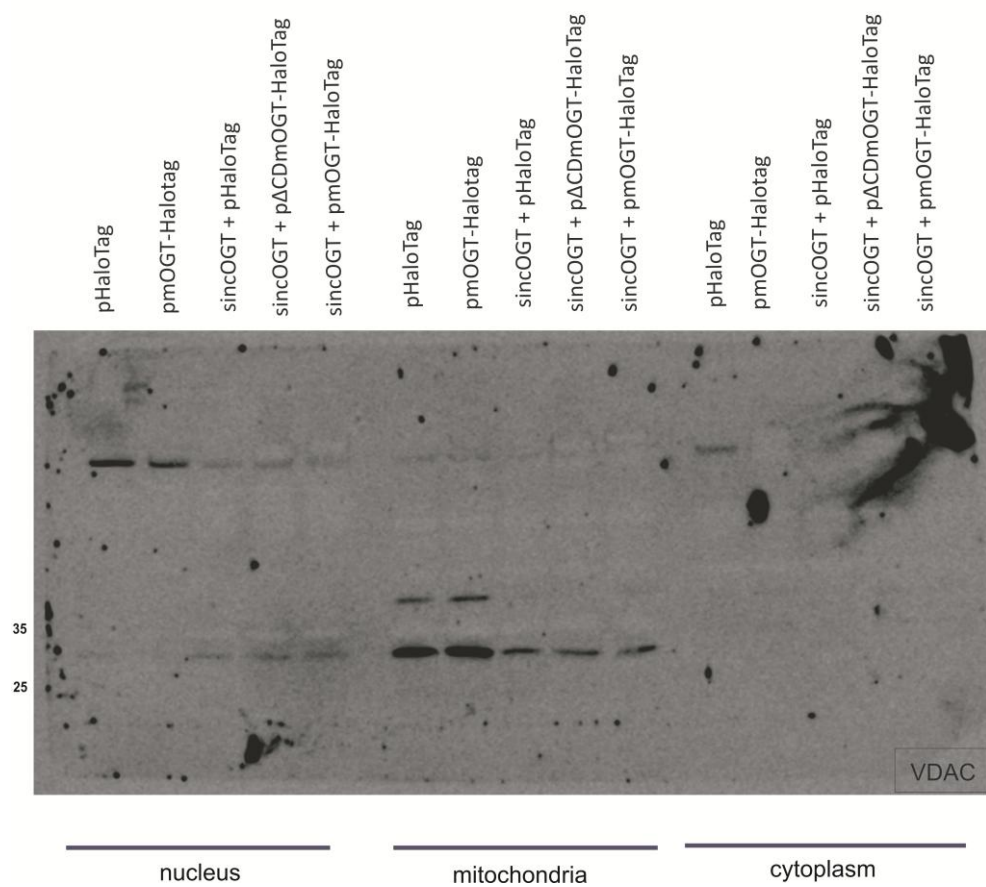

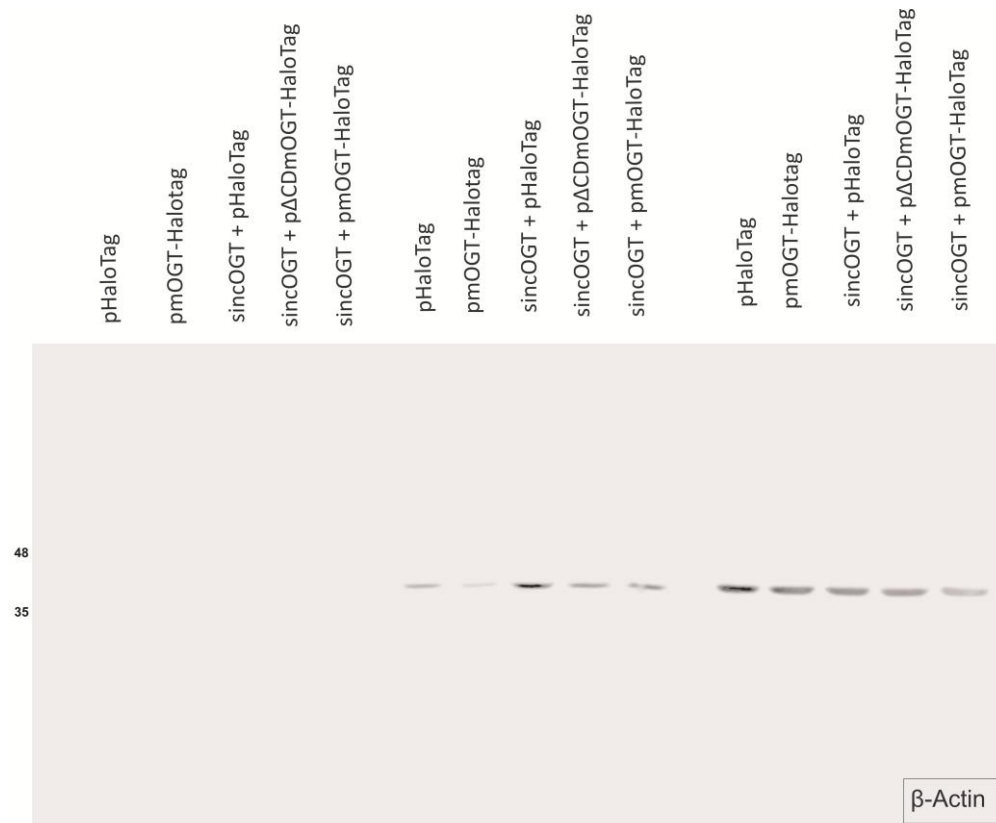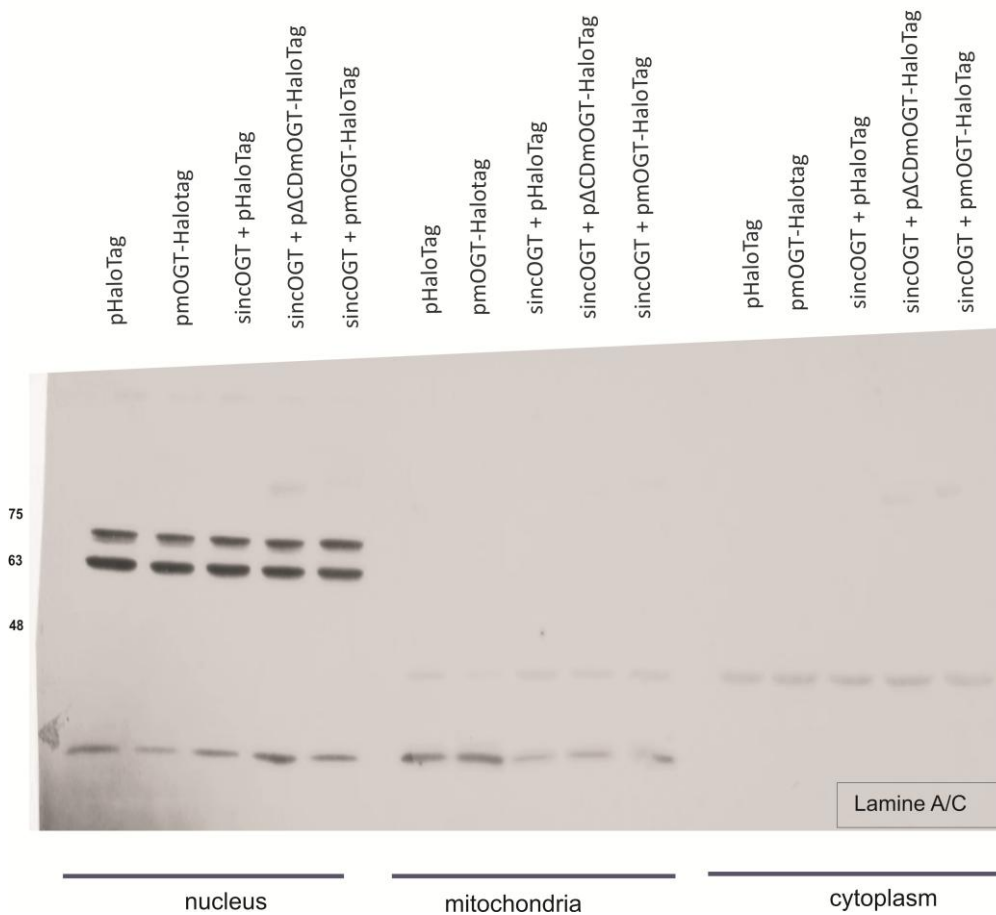

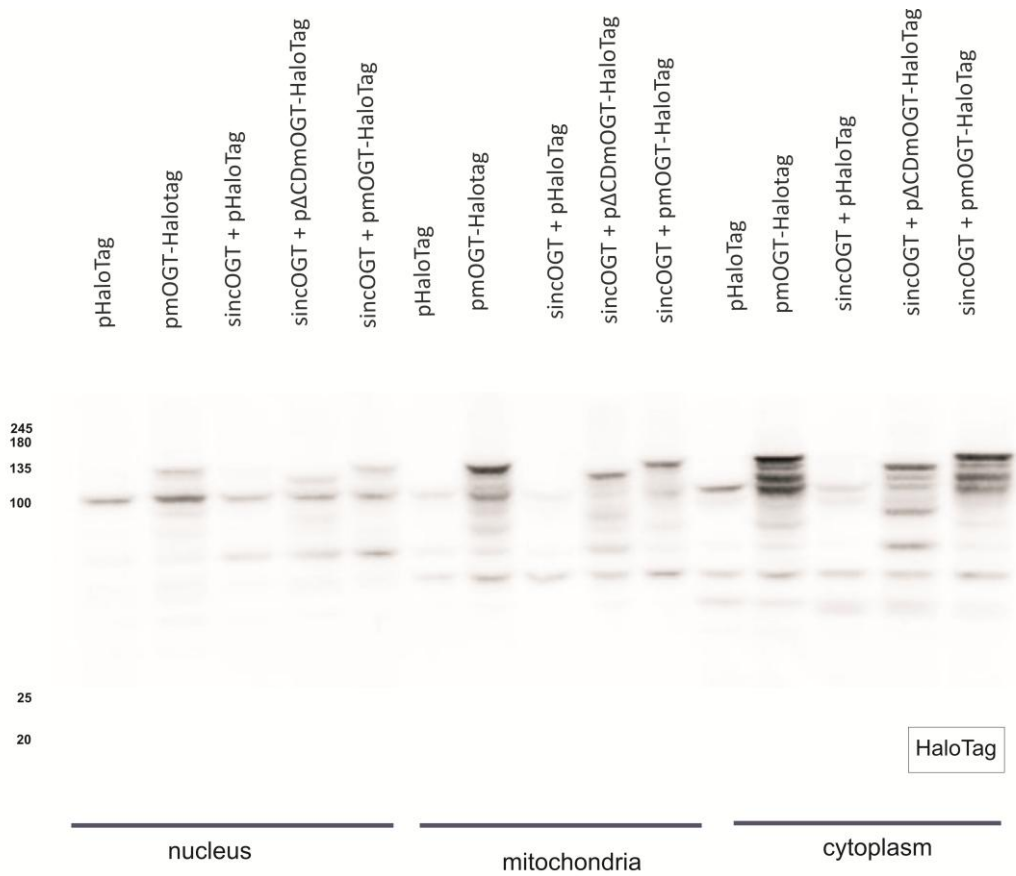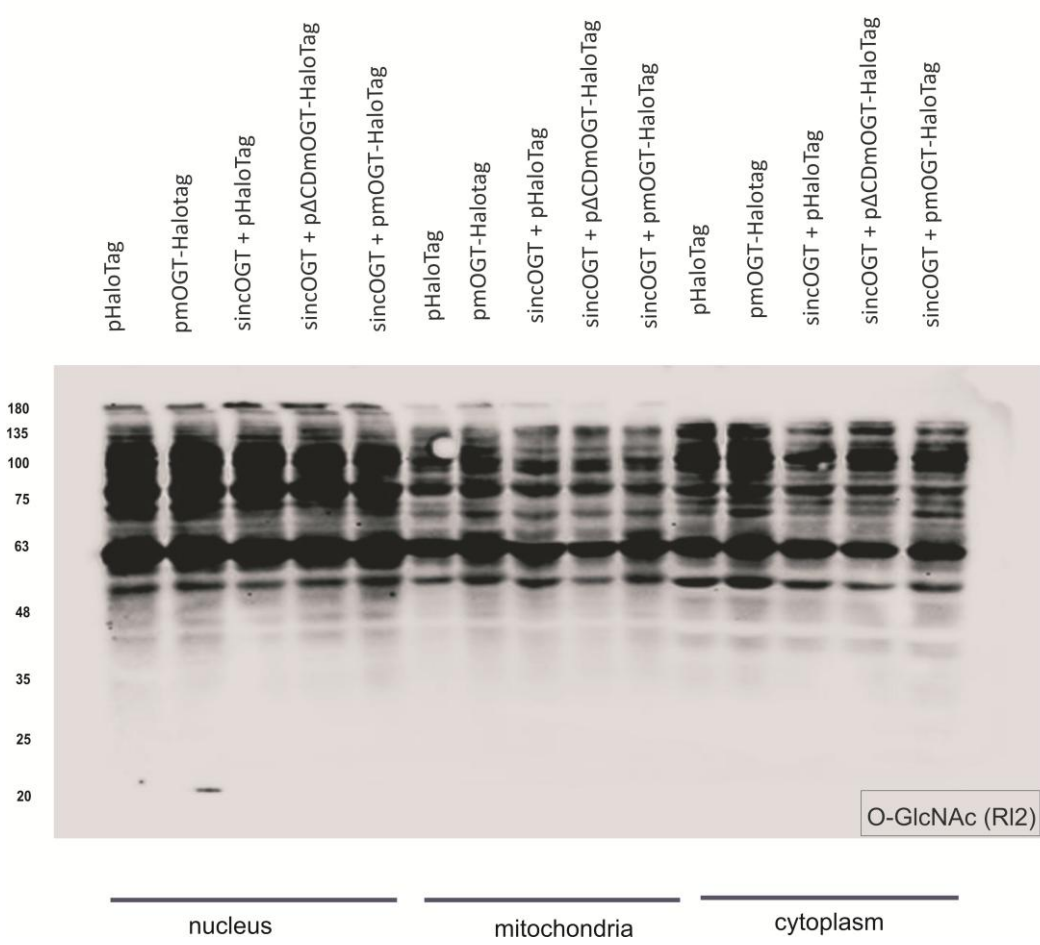

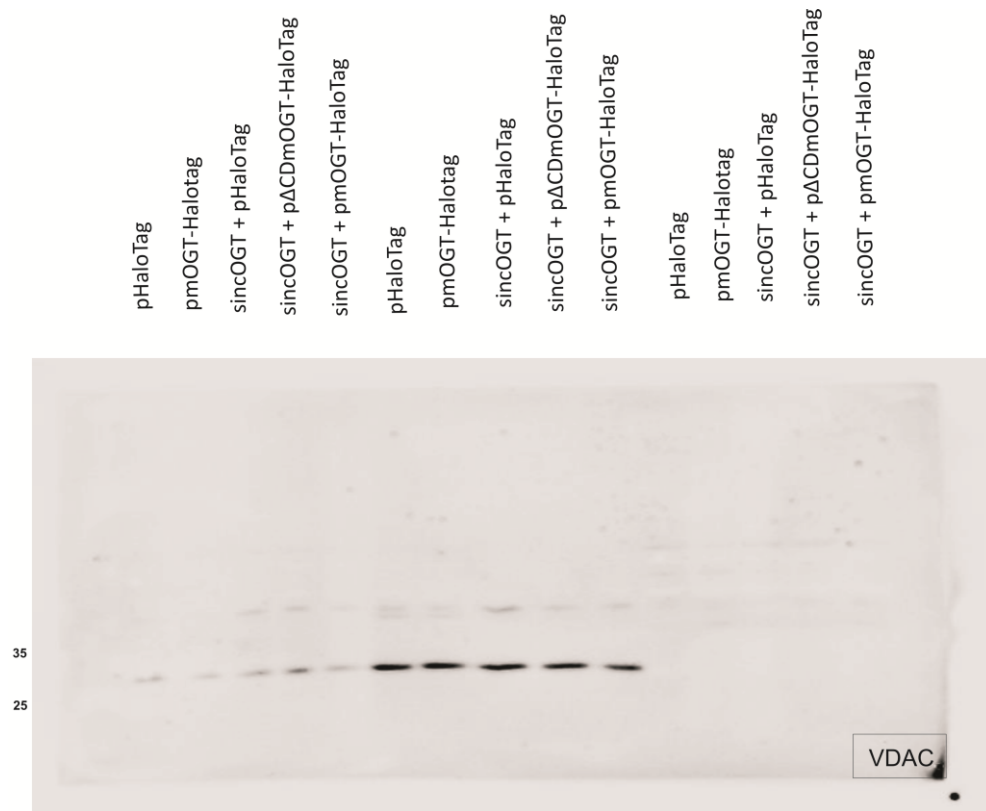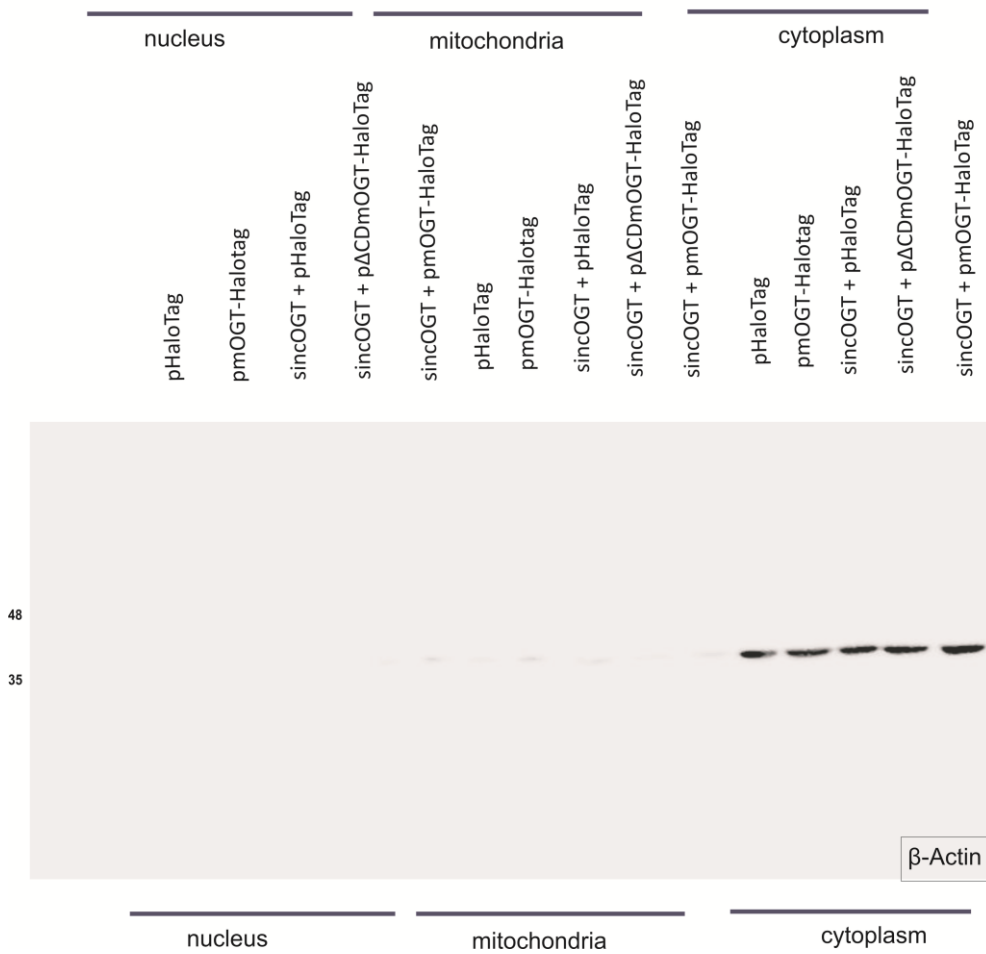

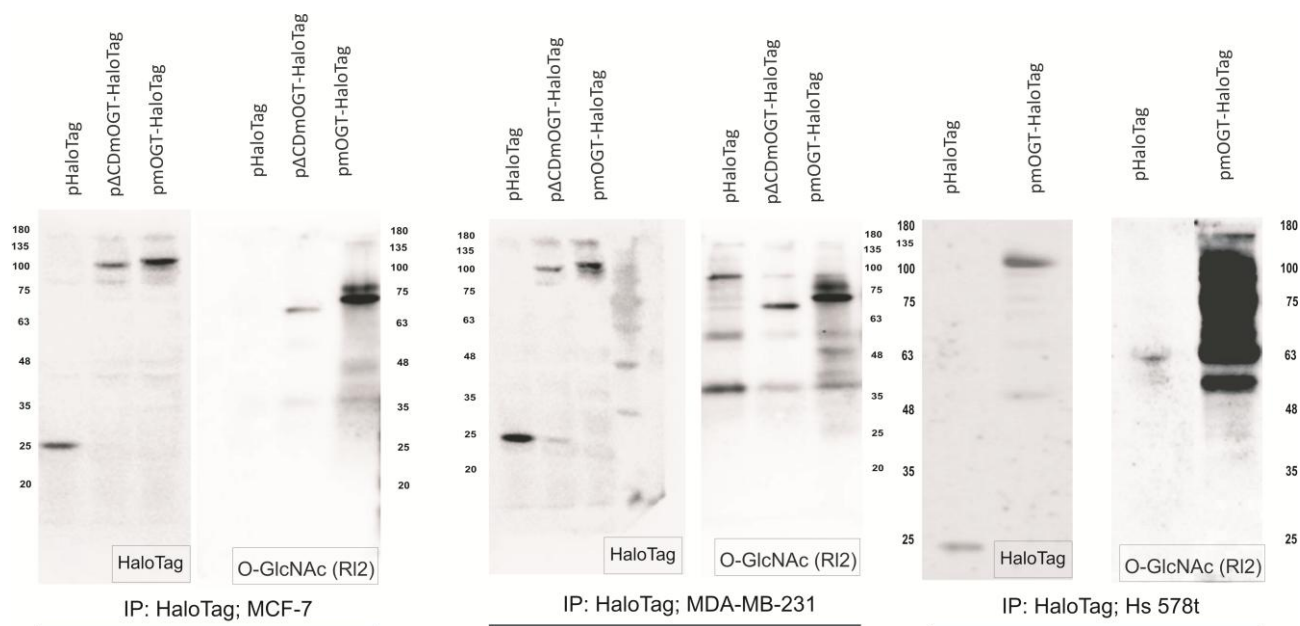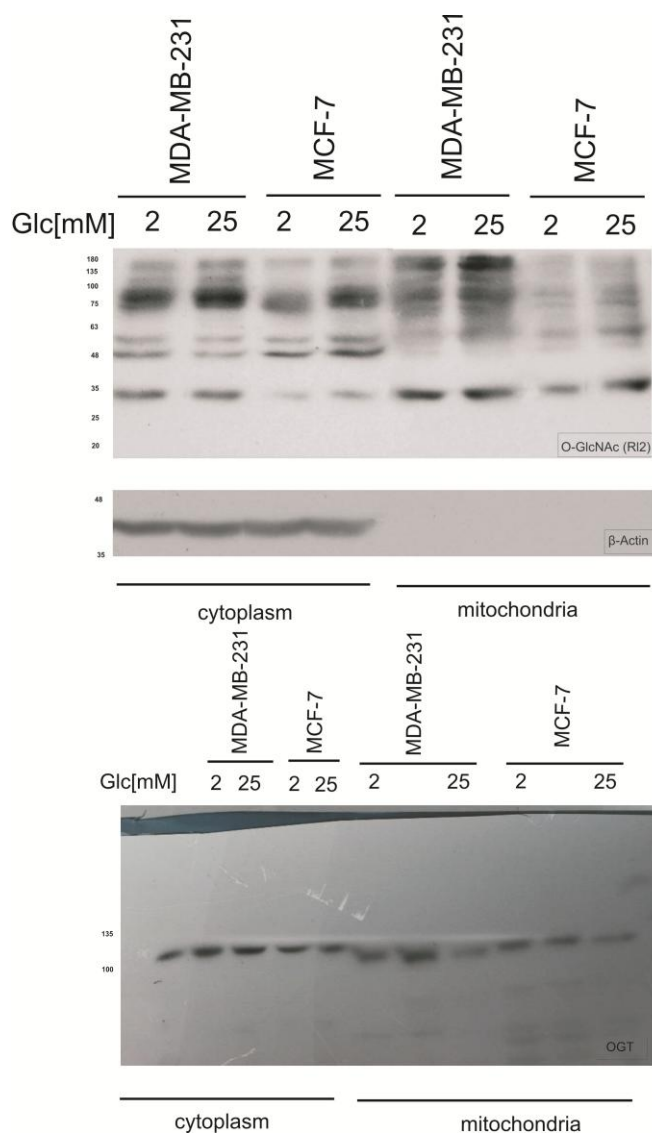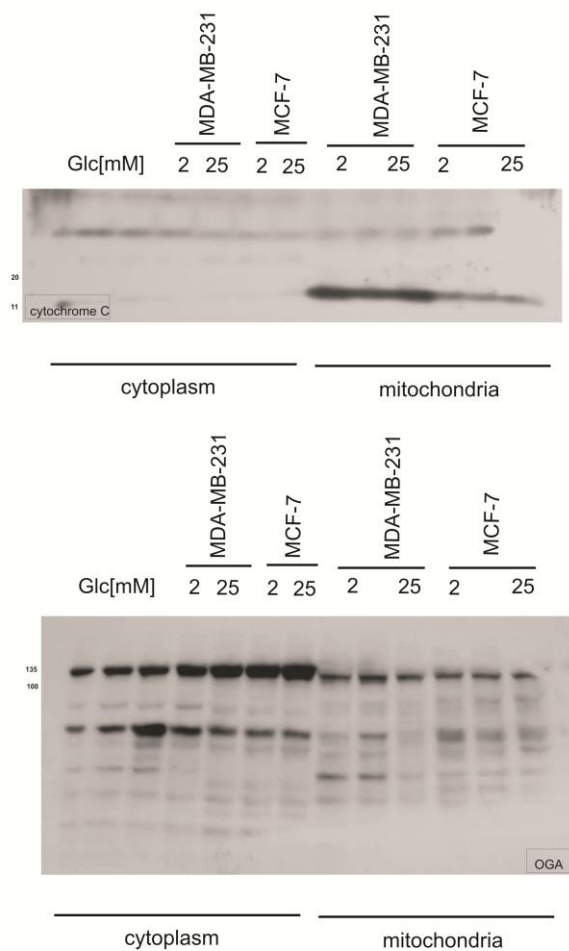

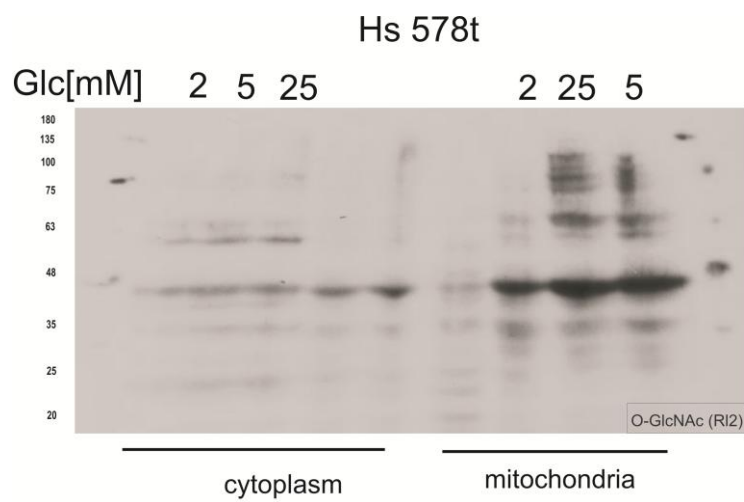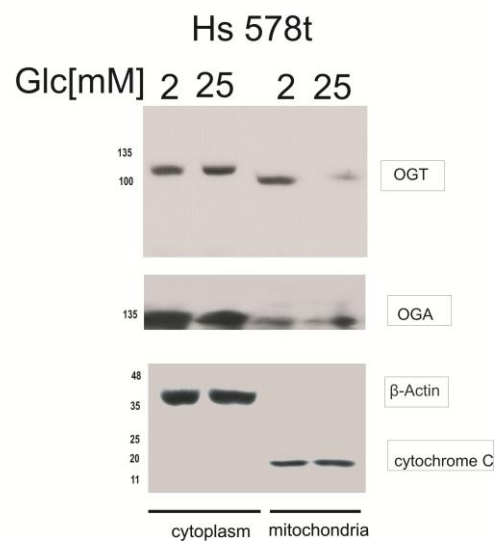

Supplement: Supplementary file 1 [file cancers-13-02956-s001.zip › File S1. The original western blot figures.pdf]
